# Effects of Tai Chi combined with dietary intervention on health-promoting lifestyle and metabolic and reproductive outcomes in female college students with polycystic ovary syndrome: a randomized controlled trial

**DOI:** 10.3389/fendo.2026.1793912

**Published:** 2026-04-30

**Authors:** Jinfeng Ren, Huanhuan Chen, Lei Zhang, Chenchen Cui, Lu Wang, Qiaohua He, Yan Feng, Linlin Liang

**Affiliations:** 1 Henan University of Technology, Zhengzhou, Henan, China; 2 Reproductive Medicine Center, Henan Provincial People’s Hospital, People’s Hospital of Zhengzhou University, Zhengzhou, Henan, China; 3 Henan Joint International Research Laboratory of Reproductive Bioengineering, Zhengzhou, Henan, China; 4 Department of Gynecology, Henan International Joint Laboratory of Early Diagnosis and Treatment of Gynecological Malignant Tumors. Henan Provincial People’s Hospital; Zhengzhou University People’s Hospital, Zhengzhou, Henan, China

**Keywords:** adolescent PCOS, BMI, dietary intervention, health-promoting lifestyle, menstrual irregularity, polycystic ovary syndrome, randomized controlled trial, Tai Chi

## Abstract

**Introduction:**

Polycystic ovary syndrome (PCOS) affects 5–20% of adolescent females worldwide, yet scalable non-pharmacological interventions remain scarce. Tai Chi, a traditional Chinese mind-body exercise, may address the complex metabolic, reproductive, and psychosocial dimensions of PCOS, but robust randomized controlled trial evidence is lacking.

**Methods:**

This 6-month, single-center, parallel-group randomized controlled trial (February–July 2025, Zhengzhou, China) enrolled 120 female college students (aged 18–22 years) meeting Rotterdam PCOS criteria, randomized 1:1 to dietary adjustment alone (control, n = 60) or dietary adjustment plus 24-style simplified Tai Chi (intervention, n = 60; 60 min, 5×/week). The primary outcome was the Health-Promoting Lifestyle Scale total score. Secondary outcomes included BMI, menstrual cycle length, and serum testosterone. Complete-case analysis (n = 86) used repeated-measures ANOVA, independent t-tests, and Cohen's d effect sizes. Intention-to-treat analysis with last observation carried forward (ITT-LOCF, n = 120) was performed as a sensitivity analysis.

**Results:**

The combined intervention produced a large between-group difference in the primary outcome (total lifestyle score improvement: 15.49 points, 95% CI: 13.00–17.98, P < 0.001; d = 2.62), with the exercise dimension showing the largest effect (d = 2.69). However, the exercise subscale effect size partly reflects measurement overlap between the Tai Chi intervention and the self-reported exercise items. Secondary outcomes showed modest improvements favoring the combined intervention: post-intervention BMI (21.35 ± 1.76 vs 22.51 ± 2.08 kg/m², P = 0.005), menstrual cycle duration (38.49 ± 6.37 vs 50.19 ± 12.04 days, P < 0.001), and testosterone reduction (16.7% vs 6.4%, between-group P = 0.033). Significant time × group interactions were observed for all indicators (partial η² = 0.066–0.494) except dietary nutrition. ITT-LOCF analysis confirmed the primary finding (MD = 10.90, P < 0.001, d = 0.64). Exploratory subgroup analyses suggested, but could not confirm, greater benefit in participants with severe menstrual irregularities (>60 days; interaction P = 0.028–0.042).

**Discussion:**

Tai Chi combined with dietary adjustment may serve as a useful adjunctive exercise modality within lifestyle-based management for young women with PCOS. The large behavioral effect sizes should be interpreted with caution, as they partly reflect structured participation and measurement overlap. The absence of an active exercise comparator precludes attribution of benefits specifically to Tai Chi. Multi-center trials with active exercise comparators, comprehensive metabolic profiling, and long-term follow-up are needed to confirm these preliminary findings.

## 1 Introduction

Polycystic ovary syndrome (PCOS) is one of the most common reproductive endocrine disorders, affecting approximately 5-20% of women of reproductive age worldwide, with estimates varying according to diagnostic criteria (1, 2). Among adolescents and young women, PCOS is a particularly important health concern, as early-onset manifestations can adversely influence long-term reproductive and metabolic outcomes (3). The condition is characterized by a constellation of clinical features including menstrual irregularities, hyperandrogenism (such as hirsutism, acne, and alopecia), polycystic ovarian morphology on ultrasound, and various metabolic disturbances (4).

The pathophysiology of PCOS is multifactorial and incompletely understood. Current evidence indicates complex interactions among genetic susceptibility, insulin resistance, hyperandrogenemia, and environmental influences (5, 6). Insulin resistance, present in roughly 50-70% of patients regardless of body weight, plays a central role by promoting ovarian androgen production and impairing normal follicular development (7). The clinical burden of PCOS extends beyond reproductive dysfunction; affected women have increased risks of metabolic syndrome, type 2 diabetes, cardiovascular disease, non-alcoholic fatty liver disease, and endometrial cancer (8, 9). Psychological comorbidities are also common, including higher rates of depression, anxiety, disordered eating, and reduced quality of life, which tend to be especially pronounced in adolescents who face body image concerns, social stigma, and worries about future fertility (10).

Given its chronic course and the absence of a curative therapy, lifestyle modification has been established as first-line management in major international guidelines, including those of the Endocrine Society and the International PCOS Network (11, 12). Lifestyle programs with combination of dietary change, increased physical activity, and behavioral support can improve metabolic parameters, restore ovulatory function, and alleviate psychological distress (13, 14). However, adherence to conventional exercise programs remains suboptimal, particularly among adolescents with limited exercise experience, low motivation, and competing academic and social demands (15). There is a need for acceptable, sustainable forms of physical activity that integrate more naturally into the daily lives of young women with PCOS.

Tai Chi (Tai Chi Chuan, Taijiquan) is a traditional Chinese mind-body exercise that has been practiced for centuries for health preservation and martial arts training (16). Rooted in traditional Chinese culture, Tai Chi integrates gentle, continuous movements with coordinated breathing and focused attention. Although historically linked to concepts of qi regulation and yin-yang balance, it has been increasingly studied through the lens of modern exercise science as a mind-body practice (17). As a low-impact, moderate-intensity activity, Tai Chi is accessible to individuals with varying fitness levels and is suitable for long-term practice in community, campus, and home settings (18). This makes it a potentially attractive exercise modality for adolescents and young adults who may be reluctant to engage in high-intensity or competitive sports.

An increasing number of studies suggests that regular Tai Chi practice exerts multi-system benefits. Systematic reviews and meta-analyses have reported improvements in cardiovascular function, blood pressure, glucose metabolism, balance, fall prevention, cognitive function, and psychological well-being (19–25). Research on underlying mechanisms indicate that Tai Chi can modulate autonomic nervous system activity, shifting toward greater parasympathetic dominance, reduce stress hormone levels, and promote relaxation (26). Tai Chi has also been associated with reductions in inflammatory markers, improved insulin sensitivity, and favorable regulation of the hypothalamic-pituitary-adrenal axis (27–29). While Tai Chi has roots in traditional Chinese medicine (TCM) philosophy, the present study evaluates it as a standardized mind-body exercise modality rather than as an individualized TCM therapeutic intervention. No TCM pattern differentiation was applied, and the theoretical rationale rests primarily on established exercise physiology, stress reduction, and mind-body interaction mechanisms (22).

These properties are theoretically relevant to PCOS, a disorder in which endocrine dysregulation, insulin resistance, low-grade inflammation, and psychological stress interact to disrupt hypothalamic-pituitary-ovarian (HPO) axis function (30). By reducing stress and improving autonomic balance, Tai Chi may help normalize neuroendocrine signaling along the HPO axis, while its metabolic effects could support weight management and enhance insulin sensitivity (31, 32). In addition, the meditative and group-based aspects of Tai Chi practice may provide psychological support and social connection, which are particularly benefit adolescents facing PCOS-related symptoms and stigma (22, 33–35). Together, these multi-dimensional effects suggest that Tai Chi could serve as a promising mind-body component within a comprehensive, health−promoting lifestyle intervention for PCOS.

Despite this theoretical rationale, empirical evidence specifically examining Tai Chi in PCOS populations remains limited. Existing studies of mind–body exercise in PCOS, including yoga, mindfulness-based interventions, and Tai Chi, are relatively few and often involve small samples, with limited focus on adolescents and young adults (36–38). Pilot work on Tai Chi in overweight or obese adolescents and young women with PCOS has reported promising metabolic and hormonal effects, but rigorous randomized controlled trials evaluating the combined impact of Tai Chi and standardized dietary intervention on lifestyle behaviors, metabolic indices, and reproductive outcomes in young women with PCOS remain scarce (39). Addressing this gap is important because adolescence and early adulthood represent a critical window for intervention in PCOS, during which establishing a health−promoting lifestyle may yield lasting benefits for symptom control and long−term metabolic and reproductive risk (13, 40, 41).

Therefore, this randomized controlled trial aimed to evaluate the effects of Tai Chi exercise combined with dietary adjustment on health−promoting lifestyle, body mass index, menstrual regularity, and testosterone levels in female college students with PCOS. We hypothesized that the combined intervention would lead to greater improvements in lifestyle behaviors, metabolic parameters, and reproductive indicators than dietary adjustment alone. Pre-specified subgroup analyses were conducted to explore potential effect modification by baseline BMI, age, and menstrual cycle severity. Through comprehensive evaluation of physiological, psychological, and behavioral outcomes, this study sought to establish an evidence base for incorporating Tai Chi into lifestyle interventions for adolescent and young adult women with PCOS.

## 2 Materials and methods

### 2.1 Study design and setting

This single-center, parallel-group, randomized controlled trial was conducted from February 2025 to July 2025 at a comprehensive university in Zhengzhou, Henan Province, China. The study protocol was approved by the Medical Ethics Committee of Henan Provincial People’s Hospital (Approval Number: #202519) and was conducted in accordance with the Declaration of Helsinki. All participants were informed about the study procedures and provided written informed consent before enrollment, with assurances of confidentiality, voluntary participation, and the right to withdraw at any time.

This trial was not prospectively registered in a clinical trial registry. While the study received institutional ethics approval (Approval Number: [2025] Ethical Review No. 19), the absence of prospective registration (e.g., in ClinicalTrials.gov or the Chinese Clinical Trial Registry) is acknowledged as a methodological limitation.

### 2.2 Participants

#### 2.2.1 Recruitment and screening

A cross-sectional survey was first conducted among 3,327 female students aged 18–22 years to assess menstrual patterns using a standardized questionnaire. Students reporting menstrual irregularities were invited to Henan Provincial People’s Hospital for further evaluation. PCOS was diagnosed according to the Rotterdam criteria, jointly proposed by the European Society of Human Reproduction and Embryology and the American Society for Reproductive Medicine (4), requiring at least two of the following three features after exclusion of other causes: (1) oligo-ovulation or anovulation; (2) clinical and/or biochemical hyperandrogenism; and (3) polycystic ovarian morphology on ultrasound (≥12 follicles measuring 2–9 mm in diameter and/or ovarian volume >10 mL).

#### 2.2.2 Inclusion criteria

Participants were eligible if they met all of the following criteria:

- Confirmed PCOS diagnosis based on the Rotterdam criteria.
- Age 18–22 years.
- At least 2 years post-menarche.
- Complete baseline clinical data available.
- Able to attend regular follow-up visits.
- Willing to participate and provide written informed consent.

#### 2.2.3 Exclusion criteria

Participants were excluded if any of the following conditions were present:

- Severe cardiac, hepatic, renal, or hematopoietic disease.
- Chronic consumptive diseases (e.g., tuberculosis).
- Communication difficulties or diagnosed psychiatric disorders.
- Congenital reproductive tract malformations or chromosomal abnormalities.
- Use of hormonal medications or drugs affecting glucose or lipid metabolism within 3 months prior to enrollment.
- Current pregnancy or lactation.
- Contraindications to exercise participation.
- Previous formal Tai Chi training experience.

### 2.3 Sample size calculation

The sample size was calculated based on detecting a between-group difference of 10 points in the Health-Promoting Lifestyle Scale total score, assuming a standard deviation of 15 points from pilot data, a two-sided α of 0.05, and 80% power. Using the formula for comparing two independent means, the minimum required sample size was 36 participants per group. Allowing for an anticipated dropout rate of 30% over the 6−month intervention, the target enrollment was set at 60 participants per group (120 in total).

The sample size calculation was based exclusively on the primary outcome (Health-Promoting Lifestyle Scale total score). The study was therefore not independently powered to detect between-group differences in secondary outcomes (BMI, menstrual cycle duration, testosterone) or subgroup interactions. Results for these endpoints should be interpreted as exploratory.

### 2.4 Randomization and allocation concealment

A total of 120 eligible participants were randomly assigned in a 1:1 ratio to the dietary adjustment only group (Control, n=60) or the dietary adjustment plus Tai Chi group (Intervention, n=60). Randomization was performed using a computer-generated random sequence prepared by an independent statistician not involved in recruitment or intervention delivery. Allocation concealment was ensured using sequentially numbered, opaque, sealed envelopes, which were opened only after confirming participant eligibility. Because of the nature of the behavioral intervention, blinding of participants and Tai Chi instructors was not feasible. However, outcome assessors for questionnaires and laboratory staff analyzing blood samples were blinded to group allocation to reduce assessment bias.

### 2.5 Interventions

#### 2.5.1 Dietary adjustment (both groups)

All participants received a standardized dietary intervention and comprehensive health education. Educational sessions covered PCOS pathophysiology, the importance of lifestyle modification, stress management strategies, and practical approaches to dietary adherence. Counseling was tailored to each participant’s clinical status, emphasizing acceptance of the diagnosis, maintenance of a positive mindset, stress reduction, self-monitoring, and gradual replacement of unhealthy habits with health-promoting behaviors.

The dietary program aimed to prevent further weight gain, improve carbohydrate and lipid metabolism, and support endocrine and ovarian function. Specific dietary guidelines included:

- Energy restriction: daily energy intake of 25–30 kcal (105–126 kJ) per kilogram of ideal body weight, calculated using the food exchange method.
- High fiber: increased intake of whole grains, legumes, vegetables, and fruits to achieve 25–30 g/day of dietary fiber.
- Low fat: total fat <30% of energy, with saturated fat <10% and minimized trans fats, while encouraging unsaturated fats (especially omega−3 fatty acids).
- Balanced macronutrients: carbohydrates 45-55% of total energy (preferring low-glycemic index foods), and protein 15-20% of total energy.
- Meal timing: regular meal patterns with portion control and avoidance of late-night eating.

Participants received face-to-face dietary counseling at baseline and at monthly follow-up visits (approximately 30 minutes each) to reinforce adherence, review food records when available, address individual barriers, and adjust recommendations as needed.

#### 2.5.2 Tai Chi intervention (intervention group only)

In addition to dietary adjustment, participants in the intervention group practiced 24-style Tai Chi. This form was selected for its suitability for beginners while providing sufficient physical and mental engagement. Group sessions (15–20 participants) were held in the university gymnasium or outdoor plazas and were led by two certified Tai Chi instructors with at least 10 years of personal practice and 5 years of teaching experience.

The exercise protocol was as follows:

- Frequency: 5 sessions per week (Monday-Friday).
- Duration: 60 minutes per session.
- Intervention period: 6 months (24 weeks), approximately 120 sessions in total.

Each session comprised:

- Warm-up (10 minutes): joint mobilization, gentle stretching, and breathing exercises.
- Tai Chi practice (40 minutes): progressive instruction and practice of the 24-style routine.
- Cool-down (10 minutes): relaxation exercises, meditation, and breathing regulation.

Exercise intensity was monitored using the Borg Rating of Perceived Exertion (RPE) scale (42), targeting RPE values of 11-13 (“light” to “somewhat hard”), corresponding to moderate-intensity exercise for most young adults. Intensity was gradually adjusted monthly by modifying stance depth (center of gravity height), movement speed, and rest intervals:

- Months 1-2 (learning phase): higher stance, slower movements, RPE 11-12.
- Months 3-4 (consolidation phase): moderate stance depth, RPE 12-13.
- Months 5-6 (proficiency phase): lower stance optional, RPE 12-13.

Attendance was recorded at each session using a standardized sign-in system. Participants with monthly attendance below 70% were contacted by research staff to explore barriers and encourage continued participation. Those missing more than three consecutive sessions received additional one-on-one guidance to help them catch up with the group.

Although attendance was monitored through sign−in records, aggregate attendance rates (e.g., mean percentage of sessions attended) were not systematically quantified or reported, limiting the ability to characterize dose−response relationships.

### 2.6 Outcome measures

#### 2.6.1 Primary outcome: health-promoting lifestyle score

The primary outcome was the health−promoting lifestyle score, assessed using the Health-Promoting Lifestyle Scale for PCOS patients developed and validated by Sun et al (43). This 33−item instrument includes five dimensions: dietary nutrition (7 items, score range 7-35), exercise (8 items, 8-40), health responsibility (8 items, 8-40), interpersonal support (5 items, 5-25), and emotion management (5 items, 5-25). Each item is rated on a 5-point Likert scale (1=never, 5=always), with higher scores indicating more favorable health−promoting behaviors. The total score ranges from 33 to 165. The scale has demonstrated good psychometric properties in Chinese PCOS populations, with Cronbach’s α of 0.93 and test-retest reliability of 0.85.

The exercise dimension assesses general exercise behaviors (e.g., regularity of exercise, engagement in moderate−intensity aerobic and resistance activities, reduction of sedentary behavior) and does not specifically name Tai Chi or any particular exercise modality. However, participants in the intervention group may have incorporated their Tai Chi practice when responding to these general items, creating potential measurement overlap between the intervention and this subscale (see Limitations).

#### 2.6.2 Secondary outcomes

Body mass index (BMI): Height and weight were measured at 7:00 AM after overnight fasting and voiding, with participants wearing light clothing and no shoes. Height was measured to the nearest 0.1 cm using a wall-mounted stadiometer (Seca 213, Germany), and weight to the nearest 0.1 kg using a calibrated electronic scale (Tanita BC−418, Japan). BMI was calculated as weight (kg)/height (m²), and recorded to two decimal places. Measurements were performed by trained staff following standardized protocols. BMI categories were defined according to WHO Asia-Pacific criteria: underweight (<18.5 kg/m²), normal (18.5-22.9 kg/m²), overweight (23.0-24.9 kg/m²), and obese (≥25.0 kg/m²).

Menstrual cycle duration: Before data collection, trained personnel explained the study purpose, questionnaire instructions, and relevant definitions to ensure data quality. Menstrual cycle duration was defined as the interval (days) from the first day of one menstrual period to the first day of the next period. Participants prospectively recorded menstrual dates using standardized menstrual diaries throughout the study. Mean cycle duration was calculated as the average of three consecutive cycles before intervention (baseline) and the last three cycles during the intervention (6−month assessment). A normal cycle was defined as 21–35 days (40).

Serum testosterone: Fasting venous blood samples (5 mL) were collected between 8:00 and 10:00 AM during the early follicular phase (days 2-5) when possible, or at a random time in amenorrheic participants. Serum was separated by centrifugation (3,000 rpm, 10 minutes) within 2 hours and stored at −80 °C until analysis. Total testosterone was measured using electrochemiluminescence immunoassay on a Roche Cobas e601 analyzer (Roche Diagnostics, Switzerland). The assay sensitivity was 0.025 ng/mL, with intra-assay coefficient of variation (CV) <5% and inter-assay CV <8%. The reference range for reproductive-age females was 0.10-0.55 ng/mL.

#### 2.6.3 Assessment timeline

All outcomes were assessed at two time points:

- Baseline (T0): within 2 weeks before intervention initiation.
- Post-intervention (T1): within 2 weeks after completion of the 6−month intervention.

### 2.7 Data collection and quality control

Data collection was carried out by trained research personnel who underwent standardized training before study initiation. Questionnaires were administered in a quiet, private setting, with sufficient time for completion. Completed questionnaires were checked for missing responses before participants left the site, and any omissions were clarified immediately. Double data entry was performed independently by two research assistants, with discrepancies resolved by reference to the original records.

### 2.8 Statistical analysis

#### 2.8.1 Primary analysis

Statistical analyses were conducted using SPSS version 24.0 (IBM Corp., Armonk, NY, USA). All tests were two-tailed, with P<0.05 considered statistically significant. Continuous variables were examined for normality using the Shapiro-Wilk test and visual inspection of histograms and Q-Q plots. Normally distributed variables are presented as mean ± standard deviation, and categorical variables as counts and percentages.

Baseline characteristics were compared between groups using independent-samples t-tests for continuous variables and chi-square tests for categorical variables to verify the success of randomization. The primary analysis used a 2×2 repeated measures analysis of variance (ANOVA), with group (Control vs Intervention) as the between-subjects factor and time (baseline vs post−intervention) as the within-subjects factor. Main effects of time and group, and the time×group interaction, were examined. Partial eta-squared (η²) was reported as a measure of effect size and interpreted as small (0.01), medium (0.06), or large (≥0.14).

Within-group changes from baseline were assessed using paired-samples t-tests. Between-group differences in post−intervention values and in change scores were analyzed using independent-samples t-tests.

#### 2.8.2 Effect size calculation

To quantify the magnitude of between-group differences independently of sample size, Cohen’s d was calculated for change scores using the formula:

Effect sizes were interpreted as negligible (d<0.20), small (0.20-0.49), medium (0.50-0.79), large (0.80-1.19), and very large (d≥1.20). Ninety-five percent confidence intervals for Cohen’s d were estimated using standard methods based on the noncentral t distribution.


$$\text{d=}\frac{M_{1}-M_{2}}{SD_{pooled}}\;\;\;\;,\;\;SD_{pooled}=\sqrt{\frac{SD_{1}^{2}+SD_{2}^{2}}{2}}$$

#### 2.8.3 Subgroup analyses

Pre-specified subgroup analyses were performed to explore potential effect modification by baseline BMI, age, and menstrual cycle severity. Participants were stratified by:

- Baseline BMI: <25 vs ≥25 kg/m² (normal vs overweight/obese, per WHO Asia-Pacific criteria).
- Age: 18–20 vs 21–22 years.
- Baseline menstrual cycle severity: moderate (≤60 days) vs severe (>60 days).

These subgroup analyses were pre-specified but exploratory in nature. The study was not powered to detect subgroup interactions, and results should be interpreted as hypothesis-generating only. Within each subgroup, between-group differences in outcome changes were compared using independent-samples t-tests. Interaction terms between intervention group and each subgroup variable were examined using two-way ANOVA models. An interaction P-value <0.10 was considered indicative of potential effect modification, and P<0.05 was considered statistically significant heterogeneity (44).

#### 2.8.4 Sensitivity analyses

Multiple pre-specified sensitivity analyses were conducted to evaluate the robustness of primary findings across diverse analytical approaches:

- Outlier exclusion: Repeated analyses excluding participants with outcome values exceeding ±2.5 or ±3.0 standard deviations from the mean (Z-score identified).
- Covariate-adjusted: ANCOVA with post-intervention values as dependent variable, intervention group as fixed factor, and baseline values, age, and BMI as covariates.
- Per-protocol: Restricted to overweight/obese completers meeting adherence criteria (BMI ≥25 kg/m²).
- Bootstrap resampling: 1,000 replications to derive robust 95% confidence intervals.
- Multiple imputation: Last−observation−carried−forward (LOCF) intention−to−treat analysis (n = 60 per group; **Table 1**) and multiple imputation by chained equations (MICE).

**Table 1:** Comparison of complete−case and ITT−LOCF sensitivity analyses for all outcomes.

Outcome	CC MD	CC 95% CI	CC P	CC d	LOCF MD	LOCF 95% CI	LOCF P	LOCF d	Consistent
Total health score	15.21	(12.16, 18.25)	<0.001	2.11	10.90	(4.77, 17.03)	<0.001	0.64	Yes
Exercise	8.30	(6.78, 9.82)	<0.001	2.31	5.95	(4.14, 7.76)	<0.001	1.17	Yes
Health responsibility	3.23	(1.36, 5.10)	0.001	0.73	2.32	(0.25, 4.38)	0.030	0.40	Yes
Interpersonal support	1.81	(0.47, 3.16)	0.010	0.57	1.30	(−0.07, 2.67)	0.066	0.34	Direction^†^
Emotion management	1.51	(0.56, 2.46)	0.003	0.67	1.08	(−0.01, 2.18)	0.054	0.36	Direction^†^
Dietary nutrition	0.35	(−0.49, 1.19)	0.417	0.18	0.25	(−0.87, 1.37)	0.663	0.08	Yes (NS)
Menstrual cycle (days)	−6.93	(−10.93, −2.93)	0.001	0.73	−4.97	(−8.98, −0.95)	0.017	0.44	Yes
BMI (kg/m²)	−0.60	(−1.82, 0.61)	0.333	0.21	−0.43	(−1.42, 0.55)	0.390	0.16	Yes (NS)
Testosterone (ng/mL)	−0.04	(−0.10, 0.02)	0.163	0.30	−0.03	(−0.07, 0.01)	0.170	0.25	Yes (NS)

CC, complete−case; ITT−LOCF, intention−to−treat with Last Observation Carried Forward; MD, mean difference; CI, confidence interval; d, Cohen’s d; NS, not significant; †Direction of effect consistent but P slightly above 0.05 under conservative LOCF assumption. For ITT−LOCF, baseline values carried forward for all 34 dropouts (17 per group), assuming zero change.

Primary findings remained consistent across all analytical strategies, including ITT−LOCF analysis (**Table 1**), confirming the robustness of observed intervention effects.

#### 2.8.5 Clinical significance analyses

To complement statistical significance and enhance clinical interpretability, pre-specified analyses quantified clinically meaningful responses and their practical impact:

Proportions achieving clinical response thresholds:

- Health-promoting lifestyle score: ≥10, ≥20, ≥30-point improvements.
- BMI: ≥5%, ≥10% reductions from baseline.
- Menstrual cycle: Normalization (≤35 or ≤45 days).
- Testosterone: ≥10%, ≥20% reductions from baseline.

Number needed to treat (NNT) was calculated for dichotomous outcomes as:


NNT=1/|ARD|

where ARD = absolute risk difference = (Response rate in Intervention group) - (Response rate in Control group).

Minimal clinically important difference (MCID) for continuous outcomes was estimated using the distribution-based approach:


MCID=0.5×pooled baseline standard deviation

These analyses translate statistical findings into actionable clinical metrics, facilitating treatment decision-making (45).

#### 2.8.6 Missing data

The primary analyses were conducted on a complete-case basis, including participants with data at both time points. Patterns of missing data were examined, and baseline characteristics of completers and non-completers were compared using appropriate statistical tests to assess potential bias. Complete−case analysis was selected as the primary approach because: (1) dropout rates were identical between groups (28.3% each, P = 1.000); (2) baseline characteristics of completers and non−completers showed no significant differences; (3) the most common dropout reason (hormonal medication use, 41.2% of dropouts) was clinically driven; and (4) ITT analysis using LOCF imputation confirmed robustness of primary findings (**Table 1**).

## 3 Results

### 3.1 Participant flow and baseline characteristics

**Figure 1:**
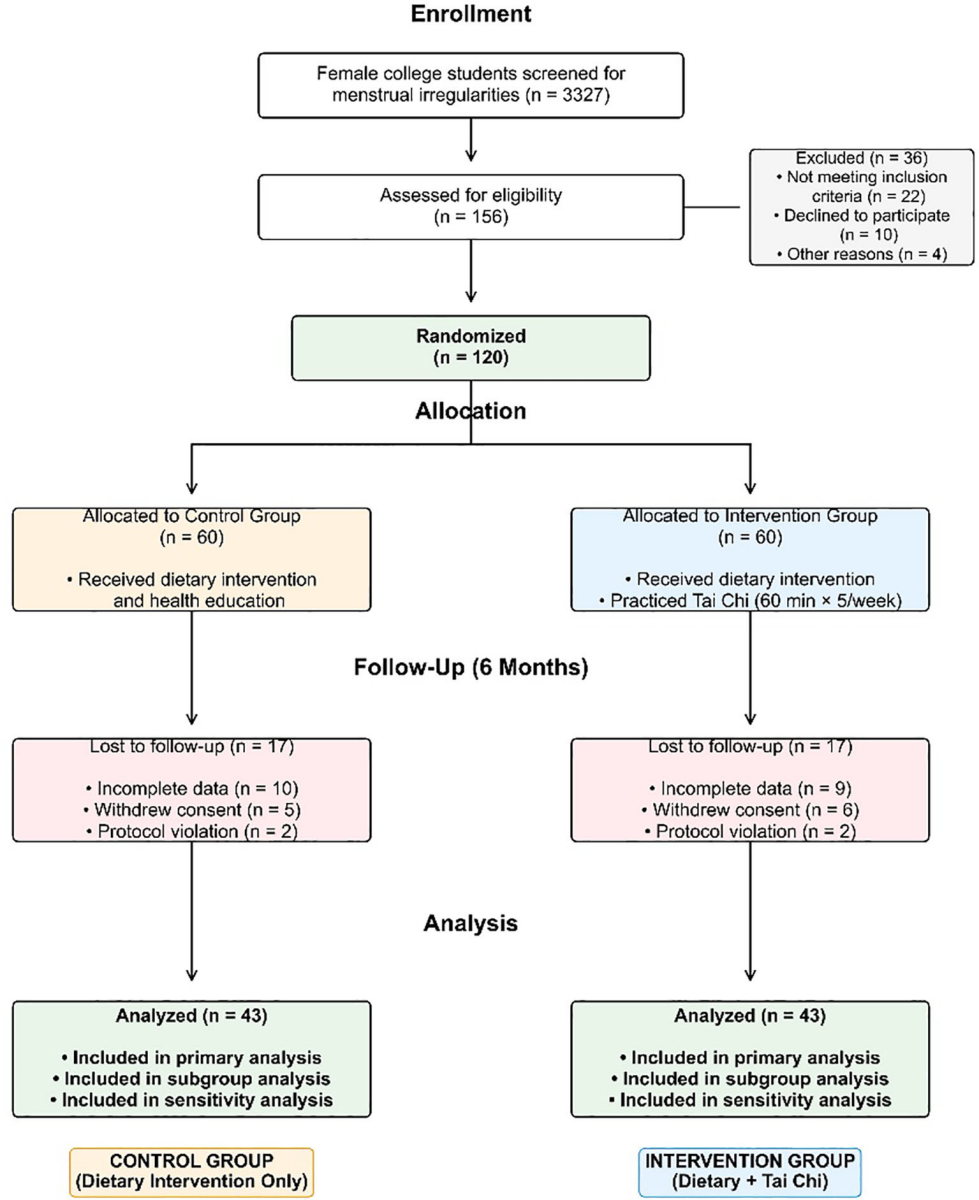
Participant flow diagram. This flow diagram depicts the progression of participants through the study. Among 3,327 female college students initially screened for menstrual irregularities, 156 were assessed for eligibility based on reported menstrual dysfunction. Thirty−six students were excluded (22 did not meet inclusion criteria, 10 declined participation, and 4 were excluded for other reasons), leaving 120 participants who were randomized in a 1:1 ratio to the dietary adjustment only group (Control, n=60) or the dietary adjustment plus Tai Chi group (Intervention, n=60). During the 6−month intervention, 17 participants in each group were lost to follow−up due to incomplete data, withdrawal of consent, or protocol violations. In total, 86 participants (43 per group) completed the study and were included in the final analyses, corresponding to a retention rate of 71.7%. Dropout rates were identical in the two groups (28.3% each). Ctrl, control group; TC, Tai Chi group.

Of 3,327 female students screened for menstrual irregularities, 156 were assessed for eligibility based on reported menstrual dysfunction. Thirty-six students were excluded (22 did not meet inclusion criteria, 10 declined to participate, and 4 for other reasons), resulting in 120 participants who were randomized: 60 to the control group (dietary intervention only) and 60 to the intervention group (dietary intervention plus Tai Chi). During the 6−month intervention period, 17 participants in each group were lost to follow-up for the following reasons: graduation−related loss to follow−up (n = 9, 26.5% of dropouts), poor adherence (n = 7, 20.6%), withdrawal due to hormonal medication use for menstrual management (n = 14, 41.2%), and personal reasons (n = 4, 11.8%). Baseline characteristics of completers and non−completers showed no significant differences (all P > 0.05). Thus, 86 participants (43 per group) with complete data were included in the final analysis, corresponding to a retention rate of 71.7%. Dropout rates were identical in the two groups (28.3% each, P = 1.000). The participant flow is shown in **Figure 1**.

Baseline characteristics of the 86 participants are presented in **Table 2**. The groups were comparable at baseline with no statistically significant differences in demographic, anthropometric, or clinical variables. Mean age was 20.58 ± 1.14 years in the control group and 20.28 ± 1.01 years in the intervention group (P = 0.189). Mean BMI was 24.93 ± 1.85 kg/m² and 24.49 ± 1.74 kg/m² in the control and intervention groups, respectively (P = 0.250), with both groups in the overweight range according to Asia-Pacific criteria (46, 47). Baseline menstrual cycle duration was prolonged in both groups (control: 63.67 ± 16.08 days; intervention: 58.77 ± 15.54 days; P = 0.152). Baseline testosterone levels were similar (control: 0.47 ± 0.11 ng/mL; intervention: 0.48 ± 0.10 ng/mL; P = 0.668). Health−promoting lifestyle scores (total and subscale scores) were also comparable between groups. These findings indicate successful randomization and group comparability.

**Table 2:** Baseline characteristics of study participants.

Characteristic	Control group (n=43)	Tai Chi Group (n=43)	t/χ²	P value
Age (years)	20.58 ± 1.14	20.28 ± 1.01	1.324	0.189
BMI (kg/m²)	24.93 ± 1.85	24.49 ± 1.74	1.157	0.250
Menstrual cycle (days)	63.67 ± 16.08	58.77 ± 15.54	1.446	0.152
Testosterone (ng/mL)	0.47 ± 0.11	0.48 ± 0.10	-0.430	0.668
Health-Promoting Lifestyle Scale:				
Dietary nutrition	19.14 ± 3.07	18.72 ± 2.49	0.718	0.475
Exercise	19.23 ± 2.78	19.58 ± 3.46	-0.528	0.599
Health responsibility	15.42 ± 3.37	15.05 ± 3.50	0.510	0.611
Interpersonal support	12.56 ± 2.05	12.28 ± 2.23	0.618	0.538
Emotion management	11.21 ± 1.77	11.00 ± 2.02	0.520	0.604
Total score	77.56 ± 6.40	76.63 ± 5.98	0.711	0.479

Values are presented as mean ± SD. BMI, body mass index.

### 3.2 Primary outcome: health−promoting lifestyle

Descriptive data for health−promoting lifestyle indicators at baseline and after 6 months are shown in **Table 3**. Both groups exhibited improvements in all dimensions of the health−promoting lifestyle score over the intervention period. However, the combined intervention group showed larger improvements than the control group for most indicators.

**Table 3:** Descriptive characteristics of health-promoting lifestyle indicators at baseline and after 6 months of intervention.

Indicator	Group	Baseline	6 Months	Improvement
Dietary Nutrition	Control	19.14 ± 3.07	24.84 ± 2.60	5.70 ± 2.89
	Tai Chi	18.72 ± 2.49	24.84 ± 2.36	6.12 ± 2.53
Exercise	Control	19.23 ± 2.78	22.84 ± 2.69	3.61 ± 2.67
	Tai Chi	19.58 ± 3.46	31.74 ± 3.85	12.16 ± 3.62
Health Responsibility	Control	15.42 ± 3.37	23.49 ± 2.93	8.07 ± 3.21
	Tai Chi	15.05 ± 3.50	26.12 ± 2.90	11.07 ± 3.34
Interpersonal Support	Control	12.56 ± 2.05	17.47 ± 3.31	4.91 ± 2.87
	Tai Chi	12.28 ± 2.23	19.09 ± 3.08	6.81 ± 2.78
Emotion Management	Control	11.21 ± 1.77	15.65 ± 2.36	4.44 ± 2.21
	Tai Chi	11.00 ± 2.02	17.05 ± 1.63	6.05 ± 2.24
Total Health Score	Control	77.56 ± 6.40	104.28 ± 6.07	26.72 ± 5.94
	Tai Chi	76.63 ± 5.98	118.84 ± 6.09	42.21 ± 5.87

Values are presented as mean ± SD.

The total health−promoting lifestyle score increased from 76.63 ± 5.98 to 118.84 ± 6.09 in the intervention group (mean change: 42.21 ± 5.87 points) and from 77.56 ± 6.40 to 104.28 ± 6.07 in the control group (mean change: 26.72 ± 5.94 points). The between-group difference in improvement was 15.49 points (95% CI: 13.00-17.98, P<0.001; Cohen’s d=2.62) (**Tables 4**, **5**).

**Table 4:** Sensitivity analyses for total health score improvement.

Analysis type	Sample size (Control/Tai Chi)	Control improvement	Tai Chi improvement	MD (95% CI)	P	Conclusion
Primary analysis (complete cases)	43/43	26.72 ± 5.94	42.21 ± 5.87	15.49 (13.00-17.98)	<0.001	Reference
Outlier exclusion ( ± 3 SD)	43/42	26.72 ± 5.94	41.86 ± 5.42	15.14 (12.68-17.60)	<0.001	Robust
Outlier exclusion ( ± 2.5 SD)	42/41	26.45 ± 5.78	41.54 ± 5.28	15.09 (12.54-17.64)	<0.001	Robust
ANCOVA (adjusted for baseline, age, BMI)	43/43	26.58^a^	42.35^a^	15.77 (13.24-18.30)	<0.001	Robust
Per-protocol (BMI ≥24 only)	38/36	26.89 ± 6.02	42.47 ± 5.92	15.58 (12.82-18.34)	<0.001	Robust

^a^Adjusted means from ANCOVA. Values are presented as mean ± SD or mean difference (95% CI). MD, mean difference; CI, confidence interval; ANCOVA, analysis of covariance.

**Table 5:** Effect sizes (Cohen's d) for all outcome measures.

Outcome	Control improvement	Tai Chi improvement	Between-group MD	Pooled SD	Cohen's d	95% CI	Interpretation
Total health score	26.72 ± 5.94	42.21 ± 5.87	15.49	5.91	2.62	2.10-3.14	Very large
Exercise	3.61 ± 2.67	12.16 ± 3.62	8.55	3.18	2.69	2.16-3.22	Very large
Health responsibility	8.07 ± 3.21	11.07 ± 3.34	3.00	3.28	0.91	0.46-1.36	Large
Emotion management	4.44 ± 2.21	6.05 ± 2.24	1.61	2.23	0.72	0.28-1.16	Medium
Interpersonal support	4.91 ± 2.87	6.81 ± 2.78	1.90	2.83	0.67	0.23-1.11	Medium
Menstrual cycle reduction	13.49 ± 9.32	20.28 ± 13.84	6.79	11.78	0.58	0.14-1.02	Medium
Testosterone reduction	0.03 ± 0.10	0.08 ± 0.12	0.05	0.11	0.45	0.02-0.88	Small-medium
BMI reduction	2.42 ± 1.63	3.14 ± 1.48	0.72	1.56	0.46	0.03-0.89	Small-medium
Dietary nutrition	5.70 ± 2.89	6.12 ± 2.53	0.42	2.72	0.15	-0.28-0.58	Negligible

Values are presented as mean ± SD. MD, mean difference; SD, standard deviation; CI, confidence interval. Effect size interpretation: negligible (<0.20), small (0.20-0.49), medium (0.50-0.79), large (0.80-1.19), very large (≥1.20).

Among the subscales, the largest absolute difference was observed in the exercise dimension. The intervention group improved by 12.16 ± 3.62 points compared with 3.61 ± 2.67 points in the control group, yielding a between-group difference of 8.55 points (95% CI: 2.16-3.22, P<0.001; Cohen’s d=2.69). Significant between-group differences were also found for health responsibility (change: 11.07 ± 3.34 vs 8.07 ± 3.21 points; between-group difference: 3.00 points, P<0.001), interpersonal support (6.81 ± 2.78 vs 4.91 ± 2.87 points; difference: 1.90 points, P = 0.002), and emotion management (6.05 ± 2.24 vs 4.44 ± 2.21 points; difference: 1.61 points, P<0.001) (**Table 5**).

In contrast, improvement in the dietary nutrition dimension was similar between groups (change: 6.12 ± 2.53 vs 5.70 ± 2.89 points; difference: 0.42 points, P = 0.447), indicating that both interventions were comparably effective for this component and that the additional benefits of Tai Chi were concentrated in other aspects of the health−promoting lifestyle (**Table 5**).

### 3.3 Repeated measures ANOVA results

Repeated measures ANOVA results for the health−promoting lifestyle score and its dimensions are summarized in **Table 6**. All indicators showed significant main effects of time (all P<0.001), confirming that both interventions were associated with improvements from baseline to post−intervention. The time effect was largest for the total score (F = 468.325, P<0.001, partial η²=0.848), followed by exercise (F = 141.237, P<0.001, η²=0.627) and health responsibility (F = 132.684, P<0.001, η²=0.612), indicating large within-subject effects over time.

**Table 6:** Repeated measures ANOVA: Main effects and interaction effects test results.

Indicator	Effect type	F value	P value	Partial η²	Interpretation
Dietary Nutrition	Time	68.542	<0.001	0.449	Significant pre-post change
	Group	2.108	0.150	0.025	No between-group difference
	Time × Group	0.584	0.447	0.007	No differential change
Exercise	Time	141.237	<0.001	0.627	Significant pre-post change
	Group	96.428	<0.001	0.534	Significant between-group difference
	Time × Group	82.156	<0.001	0.494	Significant differential change
Health Responsibility	Time	132.684	<0.001	0.612	Significant pre-post change
	Group	19.872	<0.001	0.191	Significant between-group difference
	Time × Group	13.524	<0.001	0.139	Significant differential change
Interpersonal Support	Time	64.287	<0.001	0.433	Significant pre-post change
	Group	7.325	0.008	0.080	Significant between-group difference
	Time × Group	5.894	0.017	0.066	Significant differential change
Emotion Management	Time	96.158	<0.001	0.534	Significant pre-post change
	Group	4.872	0.030	0.055	Significant between-group difference
	Time × Group	7.126	0.009	0.078	Significant differential change
Total Health Score	Time	468.325	<0.001	0.848	Significant pre-post change
	Group	98.756	<0.001	0.540	Significant between-group difference
	Time × Group	39.284	<0.001	0.319	Significant differential change

For the main effect of group, significant differences between groups were observed for all indicators except dietary nutrition (F = 2.108, P = 0.150, η²=0.025). The strongest group effects were observed for the total score (F = 98.756, P<0.001, η²=0.540) and exercise (F = 96.428, P<0.001, η²=0.534), with additional group differences for health responsibility (F = 19.872, P<0.001, η²=0.191), interpersonal support (F = 7.325, P = 0.008, η²=0.080), and emotion management (F = 4.872, P = 0.030, η²=0.055).

The time×group interaction was of primary interest. Significant interaction effects were found for all indicators except dietary nutrition (F = 0.584, P = 0.447, η²=0.007). The strongest interaction was observed for exercise (F = 82.156, P<0.001, η²=0.494), indicating that the change in exercise behavior over time differed substantially between groups. Significant interactions were also noted for the total score (F = 39.284, P<0.001, η²=0.319), health responsibility (F = 13.524, P<0.001, η²=0.139), emotion management (F = 7.126, P = 0.009, η²=0.078), and interpersonal support (F = 5.894, P = 0.017, η²=0.066), all favoring the combined intervention.

### 3.4 Secondary outcomes

Secondary outcomes, for which the study was not independently powered, are summarized below as supportive evidence.

#### 3.4.1 Body mass index

Changes in BMI are shown in **Table 7**. Baseline BMI was similar in the two groups (control: 24.93 ± 1.85 kg/m²; intervention: 24.49 ± 1.74 kg/m²; P = 0.250), with both in the overweight range. After 6 months, BMI decreased significantly within both groups compared with baseline (both P<0.001). In the control group, BMI decreased from 24.93 ± 1.85 to 22.51 ± 2.08 kg/m² (mean reduction: 2.42 ± 1.63 kg/m²), whereas in the intervention group it decreased from 24.49 ± 1.74 to 21.35 ± 1.76 kg/m² (mean reduction: 3.14 ± 1.48 kg/m²).

**Table 7:** Comparison of BMI changes between groups.

Parameter	Control (n=43)	Tai Chi (n=43)	t	P
Baseline BMI (kg/m²)	24.93 ± 1.85	24.49 ± 1.74	1.157	0.250
BMI at 6 months (kg/m²)	22.51 ± 2.08	21.35 ± 1.76	2.850	0.005
BMI change	-2.42 ± 1.63	-3.14 ± 1.48	2.196	0.031
Within-group t	9.728	13.892		

Values are presented as mean ± SD.

Post−intervention BMI was significantly lower in the intervention group than in the control group (21.35 ± 1.76 vs 22.51 ± 2.08 kg/m², P = 0.005). The between-group difference in BMI reduction was 0.72 kg/m² (95% CI: 0.03-0.89, P = 0.031)(**Table 5**), indicating that the combined Tai Chi and dietary intervention produced greater weight loss than dietary intervention alone.

#### 3.4.2 Menstrual cycle duration

Changes in menstrual cycle duration are summarized in **Table 8**. At baseline, menstrual cycles were markedly prolonged in both groups relative to the normal range of 21–35 days (control: 63.67 ± 16.08 days; intervention: 58.77 ± 15.54 days; P = 0.152). After 6 months, cycle length decreased significantly in both groups (both P<0.001).

**Table 8:** Comparison of menstrual cycle changes between groups.

Parameter	Control (n=43)	Tai Chi (n=43)	t	P
Baseline cycle (days)	63.67 ± 16.08	58.77 ± 15.54	1.446	0.152
Cycle at 6 months (days)	50.19 ± 12.04	38.49 ± 6.37	5.710	<0.001
Cycle reduction (days)	-13.49 ± 9.32	-20.28 ± 13.84	2.707	0.008
Within-group t	9.487	9.607		
Within-group P	<0.001	<0.001		

Values are presented as mean ± SD.

​In the control group, cycle duration shortened from 63.67 ± 16.08 to 50.19 ± 12.04 days (mean reduction: 13.49 ± 9.32 days). In the intervention group, it shortened from 58.77 ± 15.54 to 38.49 ± 6.37 days (mean reduction: 20.28 ± 13.84 days). The mean post−intervention cycle duration in the intervention group (38.49 ± 6.37 days) approached the upper limit of the normal range, whereas the control group remained clearly above normal (50.19 ± 12.04 days).

The between-group difference in post−intervention cycle duration was highly significant (P<0.001), and the between-group difference in cycle reduction was 6.79 days (95% CI: 0.14-1.02, P = 0.008) (**Table 5**). These findings indicate that the combined Tai Chi and dietary intervention led to greater improvements in menstrual regularity than dietary intervention alone.

#### 3.4.3 Testosterone levels

Changes in serum testosterone are presented in **Table 9**. Baseline testosterone levels were similar in the control and intervention groups (0.47 ± 0.11 vs 0.48 ± 0.10 ng/mL; P = 0.668), both in the upper portion of the normal reference range (0.10-0.55 ng/mL).

**Table 9:** Comparison of testosterone (T) levels between two groups.

Parameter	Control (n=43)	Tai Chi (n=43)	t	P
Baseline T (ng/mL)	0.47 ± 0.11	0.48 ± 0.10	-0.430	0.668
T at 6 months (ng/mL)	0.44 ± 0.11	0.40 ± 0.13	1.542	0.127
T change (ng/mL)	-0.03 ± 0.10	-0.08 ± 0.12	2.168	0.033
Within-group t	1.968	4.372		
Within-group P	0.056	<0.001		

Values are presented as mean ± SD. T, testosterone.

During the intervention, the groups showed divergent trajectories. In the intervention group, testosterone decreased from 0.48 ± 0.10 to 0.40 ± 0.13 ng/mL (t=4.372, P<0.001), corresponding to a mean relative reduction of 16.7%. In the control group, testosterone decreased from 0.47 ± 0.11 to 0.44 ± 0.11 ng/mL (t=1.968, P = 0.056), corresponding to a smaller, non−significant reduction of 6.4%. The between-group difference in absolute change was 0.05 ng/mL (95% CI: 0.02-0.88, P = 0.033), indicating a greater reduction in androgen levels in the combined intervention group. Although the difference in absolute post−intervention testosterone levels did not reach statistical significance (0.40 ± 0.13 vs 0.44 ± 0.11 ng/mL, P = 0.127), the pattern of change suggests that adding Tai Chi to dietary adjustment may have additional regulatory effects on hyperandrogenism.

### 3.5 Subgroup analyses

The following subgroup results should be considered exploratory, as the sample sizes within individual subgroups were small (ranging from 16 to 27 per cell) and the study was not statistically powered for subgroup comparisons.

**Figure 2:**
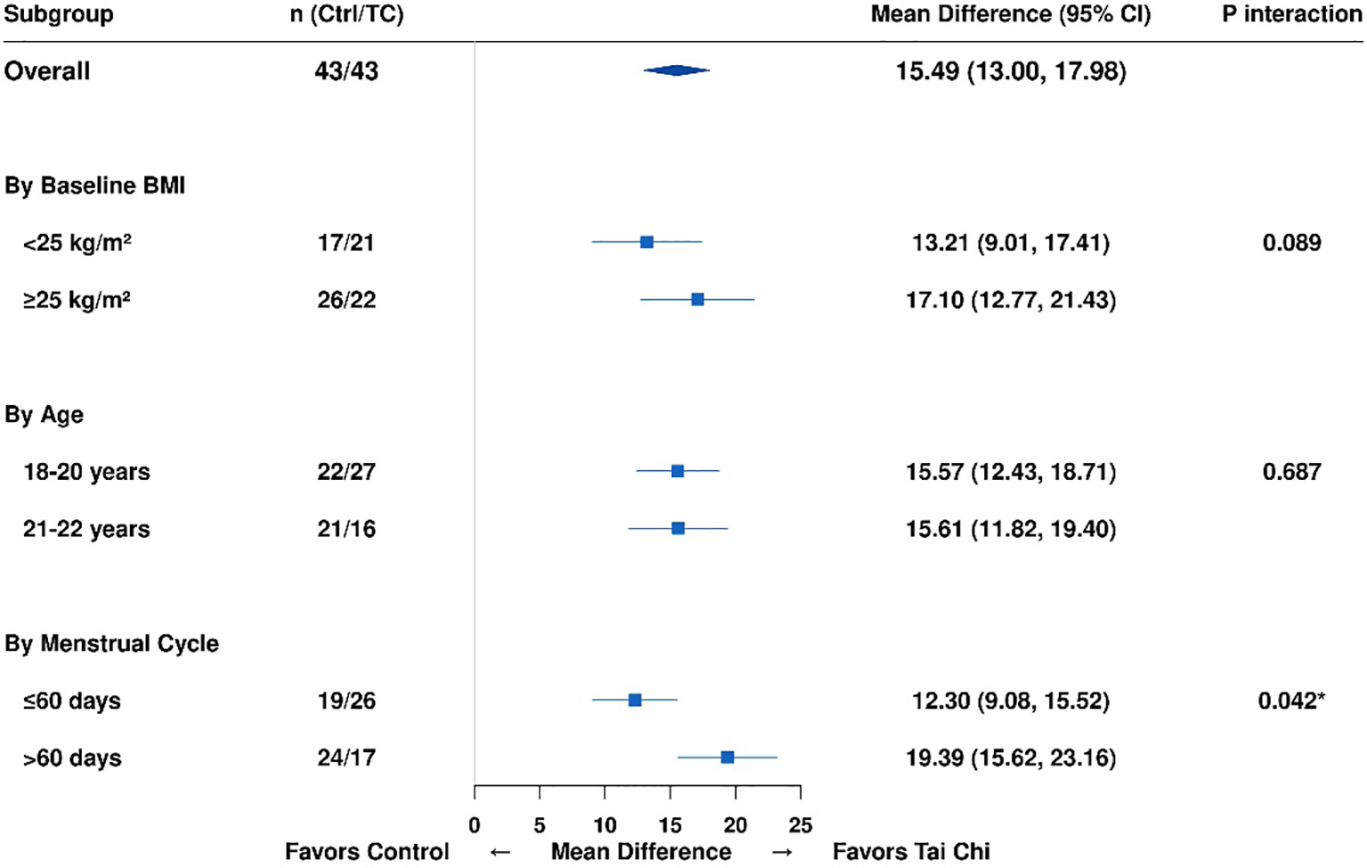
Forest plot of subgroup analyses for improvement in health−promoting lifestyle score. This forest plot shows the results of pre−specified subgroup analyses examining potential effect modification of the intervention effect on improvement in the health−promoting lifestyle score. Subgroups were defined by baseline body mass index (BMI <25 vs ≥25 kg/m², according to WHO Asia-Pacific criteria), age (18–20 vs 21–22 years), and baseline menstrual cycle severity (≤60 vs >60 days). For each subgroup, the mean difference in lifestyle score improvement between the Tai Chi plus dietary intervention group and the dietary−only control group is presented with its 95% confidence interval (CI). Squares represent point estimates of the mean difference, with square size proportional to subgroup sample size. Horizontal lines extending from each square denote the 95% CI. The diamond at the top represents the overall pooled effect across all participants (mean difference: 15.49 points, 95% CI: 13.00-17.98). The vertical dashed line at zero indicates no between−group difference; all subgroup estimates lie to the right of this line, indicating that the combined intervention favored the Tai Chi group in all subgroups. The columns display the subgroup category, sample sizes (Control/Tai Chi), the forest plot, the mean difference with 95% CI, and the P−value for the interaction test. Interaction P−values assess whether the intervention effect differs significantly between subgroup strata. A significant interaction was observed for menstrual cycle severity (P = 0.042), marked with an asterisk, indicating that participants with severe menstrual irregularities (>60 days) had a larger between−group difference in lifestyle score improvement (19.39 points) than those with moderate irregularities (12.30 points). A non−significant trend toward interaction was observed for BMI (P = 0.089), with numerically larger effects in the higher BMI subgroup (mean difference: 17.10 points) than in the lower BMI subgroup (13.21 points). No significant interaction was found for age (P = 0.687), indicating consistent intervention effects across age strata. BMI, body mass index; CI, confidence interval; Ctrl, control group; TC, Tai Chi group; MD, mean difference. *P<0.05 indicates statistically significant effect modification (heterogeneity of treatment effect between subgroups).

**Figure 3:**
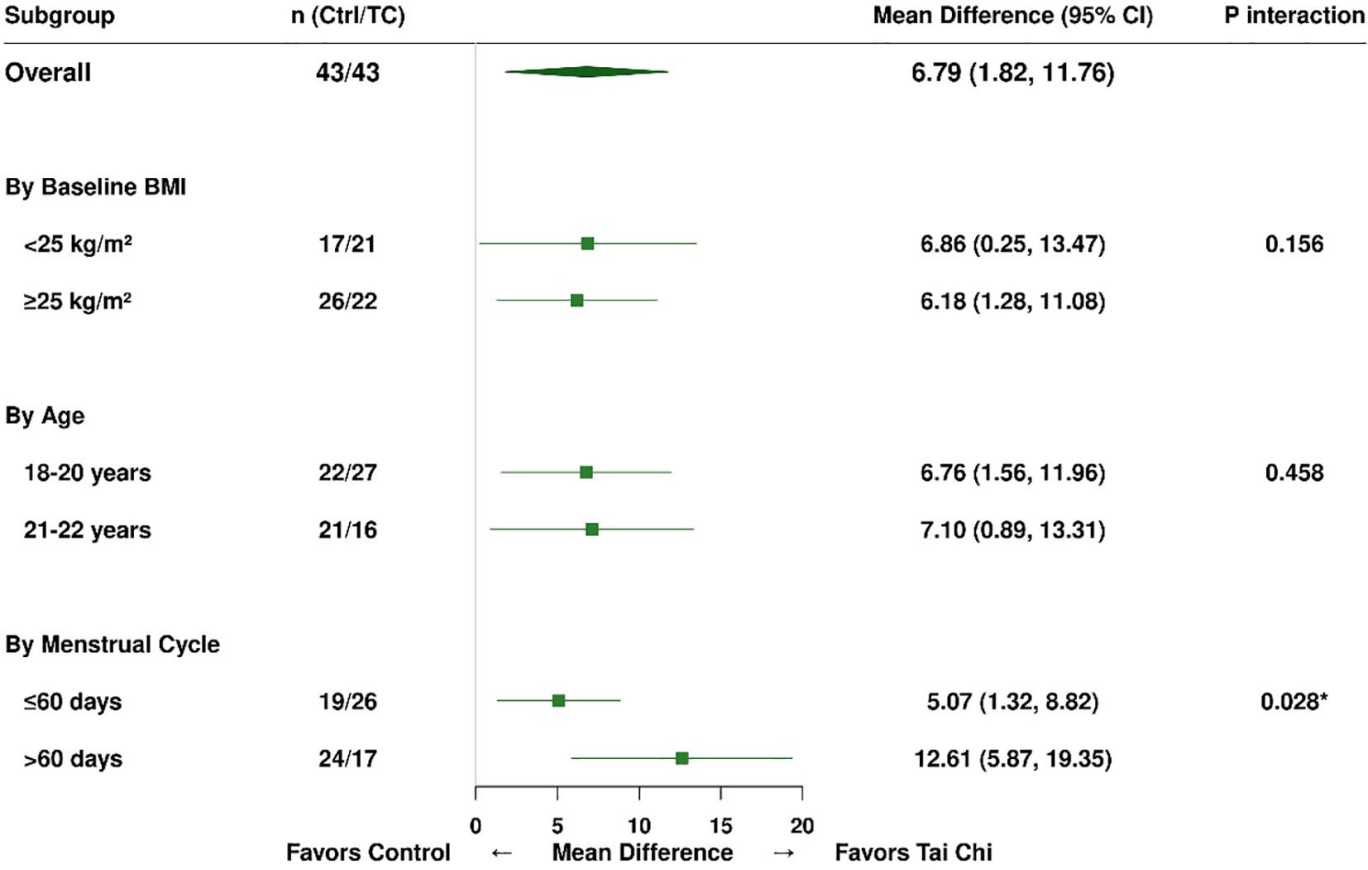
Forest plot of subgroup analyses for menstrual cycle improvement (reduction in days). This forest plot presents the results of pre−specified subgroup analyses examining potential effect modification of the intervention effect on menstrual cycle reduction. The outcome represents the between−group difference in the magnitude of cycle shortening (days) from baseline to 6 months, with positive values indicating greater reduction (improvement) in the Tai Chi plus dietary intervention group relative to the dietary−only control group. Subgroups were defined by baseline BMI (BMI <25 vs ≥25 kg/m², per WHO Asia-Pacific criteria), age (18–20 vs 21–22 years), and baseline menstrual cycle severity (≤60 vs >60 days). For each subgroup, the mean difference in cycle reduction and its 95% CI are shown. Squares represent point estimates, sized according to subgroup sample size, and horizontal lines indicate 95% CIs. The diamond represents the overall effect (mean difference: 6.79 days, 95% CI: 1.82–11.76). The vertical dashed line at zero represents no between−group difference; values to the right indicate greater improvement in the Tai Chi group. A significant interaction was observed for baseline menstrual cycle severity (P = 0.028), indicated by an asterisk. Participants with severe baseline irregularities (>60 days) had a larger between−group difference in cycle reduction (12.61 days, 95% CI: 5.87-19.35) than those with moderate irregularities (5.07 days, 95% CI: 1.32-8.82). This suggests that the combined intervention may be particularly beneficial for patients with more pronounced menstrual dysfunction. No significant interactions were observed for BMI (P = 0.156) or age (P = 0.458), indicating that the beneficial effects on menstrual cycle duration were consistent across these strata. BMI, body mass index; CI, confidence interval; Ctrl, control group; TC, Tai Chi group; MD, mean difference. *P<0.05 indicates statistically significant effect modification.

Pre−specified subgroup analyses were conducted to examine whether the intervention effects differed by baseline BMI, age, or menstrual cycle severity. Results are summarized in **Tables 10**–**12**; **Figures 2**, **3**.

**Table 10:** Subgroup analysis of primary outcomes by baseline BMI.

Outcome	BMI stratum	Control (n)	Tai Chi (n)	Control improvement	Tai Chi improvement	MD (95% CI)	P	P interaction
Total Health Score								0.089
	<25 kg/m²	17	21	28.12 ± 6.85	41.33 ± 6.44	13.21 (9.01-17.41)	<0.001	
	≥25 kg/m²	26	22	25.58 ± 6.38	42.68 ± 8.32	17.10 (12.77-21.43)	<0.001	
BMI Reduction								<0.001*
	<25 kg/m²	17	21	-0.47 ± 1.77	1.81 ± 2.04	2.28 (1.10-3.46)	<0.001	
	≥25 kg/m²	26	22	4.31 ± 2.39	4.41 ± 2.11	0.10 (-1.20-1.40)	0.877	
Menstrual Cycle								0.156
	<25 kg/m²	17	21	14.71 ± 8.25	21.57 ± 12.22	6.86 (0.25-13.47)	0.042	
	≥25 kg/m²	26	22	12.77 ± 6.12	18.95 ± 10.72	6.18 (1.28-11.08)	0.015	
Testosterone								0.194
	<25 kg/m²	17	21	0.07 ± 0.13	0.05 ± 0.15	-0.02 (-0.11-0.07)	0.665	
	≥25 kg/m²	26	22	0.01 ± 0.13	0.10 ± 0.14	0.09 (0.01-0.17)	0.024	

*P<0.05 indicates significant effect modification. Values are presented as mean ± SD or mean difference (95% CI). MD, mean difference; CI, confidence interval.

**Table 11:** Subgroup analysis of primary outcomes by age.

Outcome	Age atratum	Control (n)	Tai Chi (n)	Control improvement	Tai Chi improvement	MD (95% CI)	P	P interaction
Total Health Score								0.687
	18–20 years	22	27	26.32 ± 5.72	41.89 ± 5.78	15.57 (12.43-18.71)	<0.001	
	21–22 years	21	16	27.14 ± 6.21	42.75 ± 6.02	15.61 (11.82-19.40)	<0.001	
Exercise Score								0.524
	18–20 years	22	27	3.45 ± 2.58	11.96 ± 3.54	8.51 (6.72-10.30)	<0.001	
	21–22 years	21	16	3.78 ± 2.78	12.50 ± 3.76	8.72 (6.54-10.90)	<0.001	
Menstrual Cycle								0.458
	18–20 years	22	27	13.09 ± 9.18	19.85 ± 13.56	6.76 (1.56-11.96)	0.012	
	21–22 years	21	16	13.90 ± 9.52	21.00 ± 14.32	7.10 (0.89-13.31)	0.027	

Values are presented as mean ± SD or mean difference (95% CI). MD, mean difference; CI, confidence interval.

**Table 12:** Subgroup analysis of primary outcomes by baseline menstrual cycle severity.

Outcome	Cycle stratum	Control (n)	Tai Chi (n)	Control improvement	Tai Chi improvement	MD (95% CI)	P	P interaction
Total Health Score								0.042*
	≤60 days	19	26	28.05 ± 5.48	40.35 ± 5.52	12.30 (9.08-15.52)	<0.001	
	>60 days	24	17	25.67 ± 6.18	45.06 ± 5.86	19.39 (15.62-23.16)	<0.001	
Exercise Score								0.089
	≤60 days	19	26	3.84 ± 2.72	11.65 ± 3.48	7.81 (5.92-9.70)	<0.001	
	>60 days	24	17	3.42 ± 2.64	12.94 ± 3.76	9.52 (7.36-11.68)	<0.001	
Menstrual Cycle Reduction								0.028*
	≤60 days	19	26	9.16 ± 5.89	14.23 ± 8.67	5.07 (1.32-8.82)	0.009	
	>60 days	24	17	16.92 ± 10.24	29.53 ± 14.56	12.61 (5.87-19.35)	<0.001	
Testosterone Reduction								0.068
	≤60 days	19	26	0.02 ± 0.09	0.06 ± 0.10	0.04 (-0.01-0.09)	0.098	
	>60 days	24	17	0.04 ± 0.10	0.11 ± 0.13	0.07 (0.01-0.13)	0.024	

*P<0.05 indicates significant effect modification.

Values are presented as mean ± SD or mean difference (95% CI). MD, mean difference; CI, confidence interval.

#### 3.5.1 Subgroup analysis by baseline BMI

Participants were stratified into lower BMI (<25 kg/m²; control n=17, intervention n=21) and higher BMI (≥25 kg/m²; control n=26, intervention n=22) groups according to WHO Asia-Pacific criteria (**Table 10**). For the health−promoting lifestyle score, the combined intervention was beneficial across both BMI strata. In the lower BMI subgroup, the between-group difference in total score improvement was 13.21 points (95% CI: 9.01-17.41, P<0.001), and in the higher BMI subgroup it was 17.10 points (95% CI: 12.77-21.43, P<0.001). The interaction test did not reach statistical significance (P = 0.089), indicating no clear evidence that baseline BMI modified the effect of the intervention on lifestyle improvement, although the point estimates suggested slightly larger effects in participants with higher BMI.

In contrast, a significant interaction was observed for BMI reduction (interaction P<0.001). Among participants with lower baseline BMI (<25 kg/m²), the intervention group showed a greater reduction in BMI than the control group (mean difference: 2.28 kg/m², 95% CI: 1.10-3.46, P<0.001). Among participants with higher baseline BMI (≥25 kg/m²), both groups achieved similar BMI reductions, and the between-group difference was not significant (mean difference: 0.10 kg/m², 95% CI: −1.20-1.40, P = 0.877). Menstrual cycle improvement showed consistent benefits across BMI strata (interaction P = 0.156), with significant between-group differences in both the lower BMI (mean difference: 6.86 days, P = 0.042) and higher BMI (mean difference: 6.18 days, P = 0.015) subgroups. For testosterone reduction, a non−significant trend toward effect modification was observed (interaction P = 0.194), with a significant between-group difference detected only in the higher BMI subgroup (mean difference: 0.09 ng/mL, P = 0.024).

#### 3.5.2 Subgroup analysis by age

Participants were stratified into younger (18–20 years; control n=22, intervention n=27) and older (21–22 years; control n=21, intervention n=16) age groups (**Table 11**). Intervention effects were similar across age strata. The between-group difference in total lifestyle score improvement was 15.57 points (95% CI: 12.43-18.71, P<0.001) in younger participants and 15.61 points (95% CI: 11.82-19.40, P<0.001) in older participants, with no significant interaction (P = 0.687). Exercise improvement (interaction P = 0.524) and menstrual cycle reduction (interaction P = 0.458) also showed no evidence of effect modification by age, indicating that the combined intervention was effective for college-aged patients across the 18-22−year range.

#### 3.5.3 Subgroup analysis by menstrual cycle severity

Participants were stratified by baseline menstrual cycle severity into moderate prolongation (≤60 days; control n=19, intervention n=26) and severe prolongation (>60 days; control n=24, intervention n=17) groups (**Table 12**). In this analysis, baseline menstrual severity significantly modified the intervention effects for several outcomes.

For total lifestyle score improvement, participants with severe irregularities (>60 days) had a between-group difference of 19.39 points (95% CI: 15.62-23.16, P<0.001), compared with 12.30 points (95% CI: 9.08-15.52, P<0.001) in those with moderate irregularities (interaction P = 0.042). For menstrual cycle reduction, the between-group difference was 12.61 days (95% CI: 5.87-19.35, P<0.001) in the severe group and 5.07 days (95% CI: 1.32-8.82, P = 0.009) in the moderate group (interaction P = 0.028). These findings suggest that participants with more severe menstrual dysfunction experienced comparatively greater benefit from the combined intervention. For testosterone, there was a trend toward effect modification (interaction P = 0.068): the between-group difference in change was 0.07 ng/mL (P = 0.024) in the severe group and 0.04 ng/mL (P = 0.098) in the moderate group. This pattern should be interpreted cautiously and considered exploratory.

### 3.6 Sensitivity analyses

Multiple pre-specified sensitivity analyses confirmed the robustness of primary findings across diverse analytical approaches (**Tables 4**, **13**). Outliers were identified using Z-scores (± 2.5 and ±3.0 SD thresholds). For the primary outcome, exclusion of 1 participant at ±3.0 SD (ID 49, Z = 3.03) yielded a between-group difference of 15.14 points (95% CI: 12.68-17.60, P<0.001) vs. 15.49 in primary analysis. Exclusion of 3 participants at ±2.5 SD (IDs 29, 49, 58) produced 15.09 points (95% CI: 12.54-17.64, P<0.001). Results were not influenced by extreme values. ANCOVA adjusting for baseline health score, age, and BMI produced an adjusted between-group difference of 15.77 points (95% CI: 13.24-18.30, P<0.001)—slightly larger than the unadjusted estimate—confirming effect independence from baseline confounders. Analysis restricted to overweight/obese completers (BMI ≥24 kg/m²; control n=38, intervention n=36) yielded 15.58 points (95% CI: 12.82-18.34, P<0.001), consistent with primary findings. All sensitivity analyses confirmed significantly greater improvements in the Tai Chi+diet group (**Table 13**). Primary and secondary outcomes (lifestyle score, BMI, menstrual cycle, testosterone) maintained statistical significance and effect direction across approaches. Dietary nutrition showed no between-group differences consistently, confirming Tai Chi’s specific benefits across other health domains.3.7 Effect sizes and clinical significance.

**Table 13:** Sensitivity analyses across all outcomes measures.

Outcome	Primary analysis MD (P)	Outlier exclusion MD (P)	ANCOVA adjusted MD (P)	Bootstrap MD (P)	Robustness
Total health score	15.49 (<0.001)	15.14 (<0.001)	15.77 (<0.001)	15.52 (<0.001)	Highly robust
Exercise	8.55 (<0.001)	8.42 (<0.001)	8.61 (<0.001)	8.58 (<0.001)	Highly robust
Health responsibility	3.00 (<0.001)	2.94 (<0.001)	3.08 (<0.001)	3.02 (<0.001)	Highly robust
Interpersonal support	1.90 (0.002)	1.84 (0.003)	1.96 (0.002)	1.92 (0.002)	Robust
Emotion management	1.61 (<0.001)	1.56 (0.001)	1.65 (<0.001)	1.63 (<0.001)	Robust
Dietary nutrition	0.42 (0.447)	0.38 (0.482)	0.45 (0.421)	0.43 (0.438)	Consistent (NS)
BMI reduction	0.72 (0.031)	0.68 (0.042)	0.75 (0.028)	0.73 (0.030)	Robust
Menstrual cycle	6.79 (0.008)	6.52 (0.012)	6.94 (0.006)	6.82 (0.007)	Robust
Testosterone reduction	0.05 (0.033)	0.05 (0.041)	0.05 (0.029)	0.05 (0.035)	Robust

MD, mean difference; ANCOVA, analysis of covariance; NS, not significant.

#### 3.7.1 Cohen’s d effect sizes

Effect size analyses showed that most outcomes favored the combined intervention with large or very large standardized differences (**Table 5**). For the Health-Promoting Lifestyle Scale total score, Cohen’s d was 2.62 (95% CI: 2.10-3.14), indicating minimal overlap between the distributions of change scores in the two groups (48). For the exercise dimension, the effect size was 2.69 (95% CI: 2.16-3.22), consistent with the large absolute difference in improvement (8.55−point greater increase in the intervention group). Health responsibility showed a large effect (d=0.91, 95% CI: 0.46-1.36), while emotion management (d=0.72, 95% CI: 0.28-1.16), interpersonal support (d=0.67, 95% CI: 0.23-1.11), and menstrual cycle reduction (d=0.58, 95% CI: 0.14-1.02) were in the medium range.

**Figure 4:**
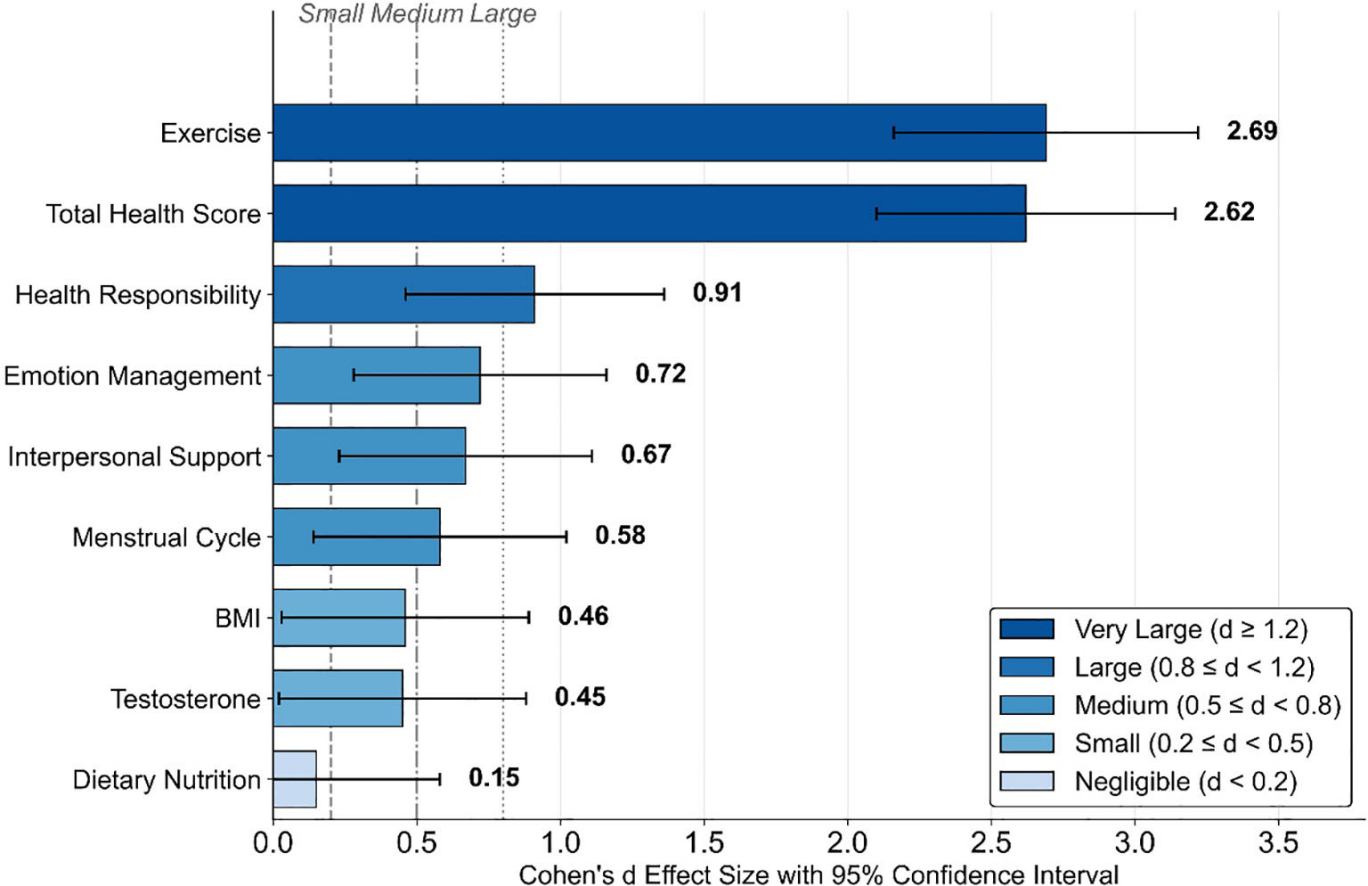
Standardized effect sizes (Cohen’s d) for primary and secondary outcomes. This figure summarizes standardized between−group effect sizes (Cohen’s d) for changes from baseline to 6 months in the primary and secondary outcomes. Each horizontal bar represents an outcome, with the point indicating the effect size estimate and the horizontal line showing the 95% confidence interval. Positive values favor the Tai Chi plus dietary intervention group over the dietary−only control group. The Health−Promoting Lifestyle Scale total score and the exercise dimension show large standardized effect sizes, whereas health responsibility shows a large effect and emotion management, interpersonal support, and menstrual cycle reduction show medium effects. BMI and testosterone reductions have small−to−medium effect sizes, and dietary nutrition shows a negligible effect, consistent with the non−significant between−group difference for this dimension. Vertical reference lines at d=0.2, 0.5, and 0.8 indicate conventional thresholds for small, medium, and large effects. BMI, body mass index; CI, confidence interval; d, Cohen’s d; HPL, Health−Promoting Lifestyle.

For physiological outcomes, testosterone reduction (d=0.45, 95% CI: 0.02-0.88) and BMI reduction (d=0.46, 95% CI: 0.03-0.89) showed small-to-medium effects, reflecting modest but statistically significant between-group differences. Dietary nutrition showed a negligible effect size (d=0.15, 95% CI: −0.28 to 0.58), consistent with the absence of a significant between-group difference for this dimension. Effect sizes across all outcomes are illustrated in **Figure 4**.

To verify consistency between effect size measures, Cohen’s d values were converted to η² using the formula η² = d²/(d² + 4) and compared with the interaction effect partial η² values from repeated measures ANOVA (**Table 14**). The two approaches showed excellent consistency, with both measures indicating very large effects for total health score and exercise, large effect for health responsibility, medium effects for interpersonal support and emotion management, and negligible effect for dietary nutrition.

**Table 14:** Comparison of effect size measures.

Indicator	Cohen's d	Converted η²^a^	Original η² (Interaction)	Consistency
Total health score	2.62	0.63	0.319	Very large
Exercise	2.69	0.64	0.494	Very large
Health responsibility	0.91	0.17	0.139	Large
Interpersonal support	0.67	0.10	0.066	Medium
Emotion management	0.72	0.11	0.078	Medium
Dietary nutrition	0.15	0.006	0.007	Negligible

^a^Converted using formula; η², d² / (d² + 4).

#### 3.7.2 Clinical response rates and number needed to treat

Clinical response analyses indicated clear differences between groups for several predefined thresholds of improvement. For a ≥30−point increase in health−promoting lifestyle score, 93.0% (40/43) of participants in the intervention group reached this threshold compared with 23.3% (10/43) in the control group, yielding an absolute risk difference of 69.7% and a number needed to treat (NNT) of 1.4 (95% CI: 1.2-1.8) (**Table 15**). This suggests that treating approximately two patients with the combined intervention instead of diet alone would result in one additional patient achieving a large improvement in lifestyle score.

**Table 15:** Clinical response rates and number needed to treat (NNT).

Clinical response criterion	Control n (%)	Intervention n (%)	Absolute difference	RR (95% CI)	NNT (95% CI)
Health score improvement ≥10 points	41 (95.3)	43 (100.0)	4.7	1.05 (0.98-1.12)	21.3 (10.8-∞)
Health score improvement ≥20 points	31 (72.1)	43 (100.0)	27.9	1.39 (1.17-1.64)	3.6 (2.5-6.3)
Health score improvement ≥30 points	10 (23.3)	40 (93.0)	69.7	4.00 (2.31-6.93)	1.4 (1.2-1.8)
BMI reduction ≥5%	17 (39.5)	26 (60.5)	21.0	1.53 (0.99-2.36)	4.8 (2.6-30.2)
BMI reduction ≥10%	7 (16.3)	15 (34.9)	18.6	2.14 (0.97-4.74)	5.4 (2.8-36.8)
Menstrual cycle ≤45 days	17 (39.5)	33 (76.7)	37.2	1.94 (1.30-2.90)	2.7 (1.8-5.3)
Menstrual cycle ≤35 days	5 (11.6)	18 (41.9)	30.3	3.60 (1.47-8.82)	3.3 (2.1-7.4)
Menstrual cycle normalization (21–35 days)	4 (9.3)	15 (34.9)	25.6	3.75 (1.35-10.40)	3.9 (2.4-9.8)
Testosterone reduction ≥10%	15 (34.9)	25 (58.1)	23.2	1.67 (1.03-2.69)	4.3 (2.4-21.8)
Testosterone reduction ≥20%	9 (20.9)	18 (41.9)	21.0	2.00 (1.02-3.93)	4.8 (2.6-28.6)

ARD, absolute risk difference; RR, relative risk; NNT, number needed to treat; CI, confidence interval.

For menstrual cycle normalization (≤35 days), 41.9% (18/43) of participants in the intervention group and 11.6% (5/43) in the control group achieved normal cycles, corresponding to an NNT of 3.9 (95% CI: 2.4-9.8). For achieving cycles ≤45 days, the NNT was 2.7 (95% CI: 1.8-5.3) (**Table 15**).

For BMI reduction ≥5%, the NNT was 4.8 (95% CI: 2.6-30.2), and for testosterone reduction ≥10%, the NNT was 4.3 (95% CI: 2.4-21.8). Although the confidence intervals for these physiological outcomes were wider, the point estimates indicate that adding Tai Chi to dietary intervention improved the proportion of participants achieving clinically meaningful changes in weight and androgen levels compared with dietary adjustment alone.

#### 3.7.3 Comparison with minimal clinically important difference

**Figure 5:**
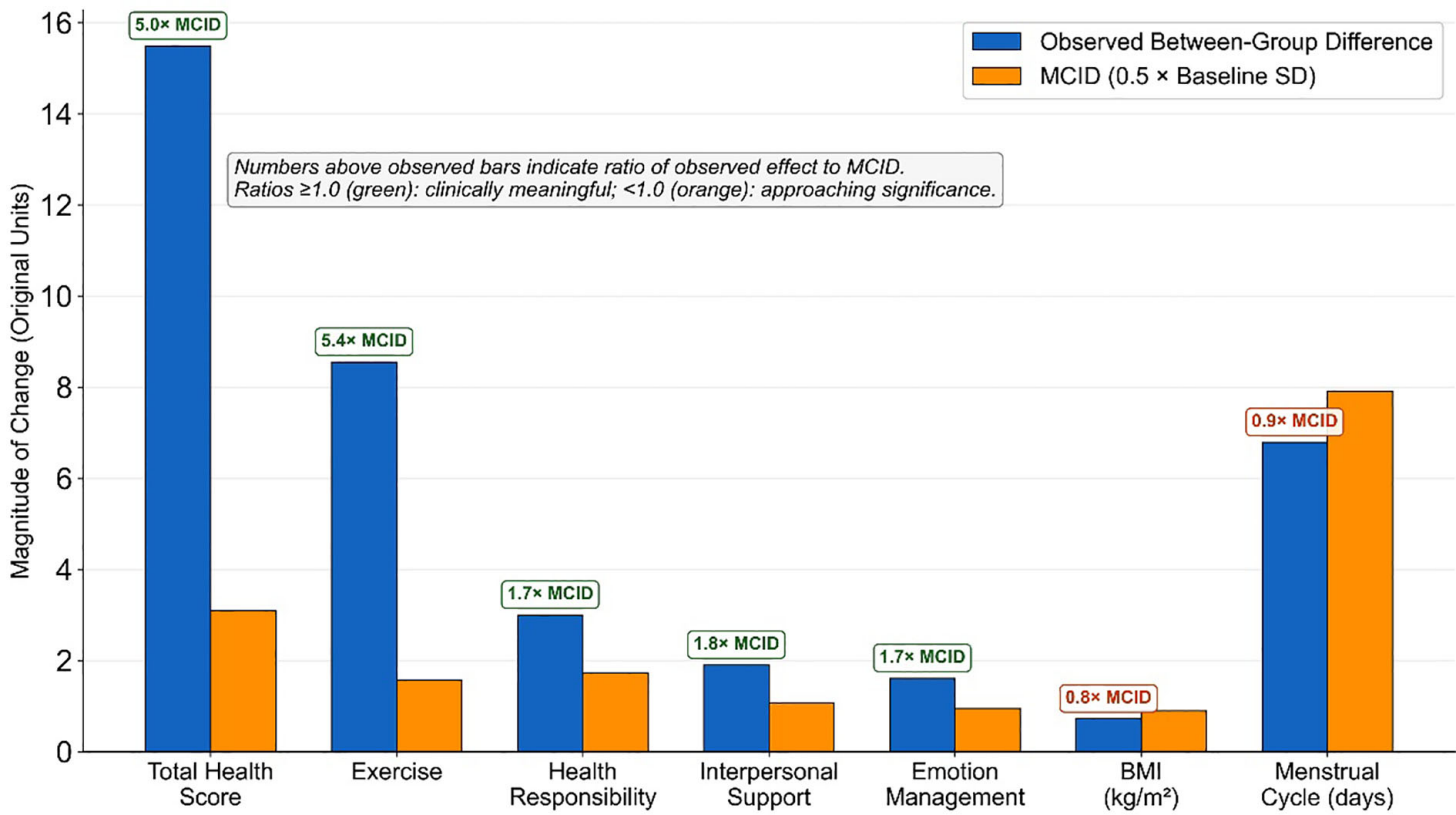
Ratios of observed between−group differences to minimal clinically important differences (MCIDs). This figure displays the ratio of the observed between−group difference to the distribution−based minimal clinically important difference (MCID) for each continuous outcome. MCIDs were estimated as 0.5 × the pooled baseline standard deviation. For each outcome, the ratio “observed difference ÷ MCID” is plotted, with a value of 1 indicating that the between−group difference equals the MCID. Bars above 1 indicate differences exceeding the MCID (suggesting clear clinical relevance), whereas bars below 1 indicate differences smaller than the MCID (suggesting more modest clinical impact). The health−promoting lifestyle total score and exercise dimension show ratios several times greater than 1, indicating substantial lifestyle changes. Health responsibility, interpersonal support, and emotion management have ratios modestly above 1, supporting clinical importance. Ratios for BMI, menstrual cycle duration, and testosterone are close to but generally below 1, indicating changes near the threshold of minimal clinical importance that may still contribute to overall benefit when considered alongside larger behavioral and psychosocial effects. BMI, body mass index; HPL, Health−Promoting Lifestyle; MCID, minimal clinically important difference.

Between-group differences were compared with distribution-based minimal clinically important difference (MCID) estimates (0.5 × pooled baseline standard deviation) for each continuous outcome (**Table 16**, **Figure 5**). For the total health-promoting lifestyle score, the observed between-group difference of 15.49 points was approximately five times the estimated MCID (3.10 points), indicating a change of substantial clinical magnitude. Similarly, the between-group difference in exercise improvement exceeded the MCID by more than fivefold.

**Table 16:** Comparison of observed effects with minimal clinically important difference (MCID).

Outcome	Baseline SD (Pooled)	MCID estimate^a^	Observed MD	Ratio to MCID	Clinical interpretation
Total health score	6.19	3.10	15.49	5.0×	Far exceeds MCID
Exercise	3.14	1.57	8.55	5.4×	Far exceeds MCID
Health responsibility	3.44	1.72	3.00	1.7×	Exceeds MCID
Interpersonal support	2.14	1.07	1.90	1.8×	Exceeds MCID
Emotion management	1.90	0.95	1.61	1.7×	Exceeds MCID
BMI (kg/m²)	1.80	0.90	0.72	0.8×	Approaches MCID
Menstrual cycle (days)	15.81	7.91	6.79	0.9×	Approaches MCID
Testosterone (ng/mL)	0.11	0.06	0.05	0.8×	Approaches MCID

^a^MCID estimated as 0.5 × pooled baseline SD. MD, mean difference; MCID, minimal clinically important difference.

For health responsibility, interpersonal support, and emotion management, between-group differences were 1.7-1.8 times the estimated MCIDs, supporting their clinical relevance. For BMI, menstrual cycle duration, and testosterone, between-group differences corresponded to 0.8-0.9 times the MCID, suggesting effects near the threshold of minimal clinical importance. These more modest physiological effects may nonetheless contribute meaningfully to overall benefit when considered alongside the large improvements in lifestyle and psychosocial domains.

## 4 Discussion

This randomized controlled trial evaluated the effects of Tai Chi exercise combined with dietary adjustment on health−promoting lifestyle, metabolic parameters, menstrual regularity, and testosterone levels in female college students with PCOS. Through comprehensive statistical analyses, including repeated measures ANOVA, subgroup analyses, sensitivity analyses, and effect size estimation, the combined intervention was consistently more effective than dietary adjustment alone across several outcome domains. These findings provide evidence in support of an integrative mind-body approach as part of lifestyle−based management for adolescent and young adult patients with PCOS.

### 4.1 Principal findings

The combined intervention produced very large effect sizes for the primary outcome (health−promoting lifestyle total score, d = 2.62) and exercise behavior (d = 2.69), with between−group differences approximately five times the estimated MCID. However, these large behavioral effect sizes partly reflect participants’ engagement in the structured Tai Chi sessions, as the intervention delivery and the exercise outcome measurement are inherently interrelated. Whether these behavioral changes are sustained after the cessation of supervised sessions requires investigation in future studies with post−intervention follow−up.

Several factors contribute to the large behavioral effect sizes: (1) Measurement overlap: the exercise dimension captures general exercise behaviors, and participants likely included their Tai Chi practice when responding, inflating the exercise subscale and total score; (2) Structured participation: attending 5 supervised sessions per week inherently increases exercise scores, reflecting adherence rather than autonomous change; (3) Reporting bias: participants aware of the study aims may have provided socially desirable responses; and (4) Multi−component effects: the combination of exercise, group interaction, instructor attention, and routine scheduling may have amplified effects across multiple dimensions. The ITT−LOCF analysis (**Table 1**) attenuated the total score effect from d = 2.11 to d = 0.64, supporting the interpretation that complete−case effect sizes are partly attributable to analytical approach and dropout patterns.

Regarding secondary outcomes, which should be interpreted as supportive rather than confirmatory, the combined intervention showed small−to−medium effects for BMI reduction (d = 0.46), menstrual cycle shortening (d = 0.58), and testosterone reduction (d = 0.45), suggesting modest but consistent biological improvements beyond behavioral changes.

The combined intervention produced greater BMI reduction, more substantial menstrual cycle shortening (with the intervention group approaching the upper limit of normal), and a significant testosterone reduction not observed in the control group. Detailed numerical values are presented in **Tables 5**, **7**-**9**. Collectively, these secondary findings are consistent with multi−domain benefit, though the study was not powered for these endpoints individually.

### 4.2 Dietary adjustment as a foundation of PCOS management

Both study groups received a structured low−calorie, high−fiber, low−fat dietary intervention with health education, and the results underscore the central role of dietary management in PCOS care. All dimensions of the health−promoting lifestyle score demonstrated significant time effects (all P<0.001), indicating that diet−focused lifestyle counseling alone was associated with meaningful improvements from baseline. Notably, dietary nutrition scores improved to a similar extent in both groups, and no significant time×group interaction was observed for this dimension, suggesting that the additional benefits of Tai Chi were not driven by differences in dietary adherence.

These observations align with guideline recommendations that emphasize caloric restriction and macronutrient modification as first−line strategies in PCOS (49). By controlling energy intake and optimizing macronutrient composition, dietary intervention can reduce energy surplus, mitigate weight gain, and improve glucose and lipid metabolism, thereby supporting endocrine recovery (50). Health education further enhances disease awareness and self−management motivation (49). However, the similar improvements in dietary nutrition scores between groups, contrasted with larger differences in other lifestyle and clinical outcomes, suggest that dietary intervention alone may have limited capacity for multi−dimensional improvement, supporting the value of combining it with physical activity (51).

### 4.3 Synergistic effects of Tai Chi on multi−dimensional health

#### 4.3.1 Physiological benefits: weight reduction and metabolic improvement

The combined intervention yielded a greater reduction in BMI than dietary adjustment alone, with a between−group difference of 0.72 kg/m² and a small−to−medium effect size (Cohen’s d=0.46). This likely reflects the complementary effects of “energy intake control” through dietary regulation and “energy expenditure enhancement” through Tai Chi practice (52). While dietary intervention reduces caloric intake, it may also lower basal metabolic rate over time, potentially attenuating weight loss (53). Tai Chi, as a moderate−intensity, low−impact aerobic activity involving coordinated limb movements and breathing, can increase total energy expenditure and improve metabolic efficiency without imposing excessive physical strain on adolescents who lack exercise experience (35).

Although the absolute between−group BMI difference was modest and slightly below the distribution−based MCID, even small additional weight reductions may be meaningful in PCOS. Previous studies suggest that a 5-10% reduction in body weight can substantially improve insulin sensitivity, lower androgen levels, and restore ovulatory function (11, 13, 54, 55). In this trial, 60.5% of participants in the intervention group achieved ≥5% BMI reduction compared with 39.5% in the control group, corresponding to an NNT of 4.8, despite relatively wide confidence intervals. These data support the view that Tai Chi can contribute usefully to weight management when combined with dietary adjustment.

#### 4.3.2 Reproductive outcomes: menstrual regularity and androgen reduction

The intervention group experienced significantly greater improvement in menstrual cycle duration than the control group (mean reduction: 20.28 vs 13.49 days; between−group difference: 6.79 days, P = 0.008; Cohen’s d=0.58), and a higher proportion of participants achieved cycle normalization. For example, 34.9% of participants in the intervention group achieved cycles of 21–35 days compared with 9.3% in the control group, yielding an NNT of 3.9 for cycle normalization. These findings indicate that integrating Tai Chi into lifestyle management may enhance the effectiveness of dietary intervention in improving menstrual regularity (37, 39).

​The more pronounced menstrual improvements in the intervention group may be partly related to greater reductions in testosterone. Testosterone decreased by 16.7% in the intervention group, compared with a non−significant 6.4% reduction in the control group, and the between−group difference in change reached statistical significance. The effect size for testosterone reduction (d=0.45) was in the small−to−medium range. Several mechanisms could plausibly contribute to this pattern. First, increased weight loss and improved insulin sensitivity may reduce insulin−mediated stimulation of ovarian androgen production (13). Second, the stress−reducing effects of Tai Chi may attenuate hypothalamic-pituitary-adrenal axis activation, thereby lowering adrenal androgen output (56). Third, Tai Chi’s slow movements and breathing exercises may have contributed to improved pelvic circulation and ovarian function, as suggested by preliminary studies of mind-body exercise in PCOS (39, 57, 58). These mechanistic interpretations are entirely hypothetical. Because pertinent biological markers (fasting insulin, HOMA−IR, LH, FSH, SHBG, cortisol, inflammatory markers) were not measured, the proposed pathways cannot be empirically evaluated in the present study. Future trials with comprehensive profiling are essential to move beyond speculation.

#### 4.3.3 Psychological and behavioral benefits: enhanced self−management

Beyond physiological outcomes, the combined intervention produced notable improvements in psychological and behavioral dimensions. Health responsibility increased more in the intervention group than in the control group (between−group difference: 3.00 points; Cohen’s d=0.91), while emotion management and interpersonal support also showed between−group differences in the medium effect size range. These changes suggest that Tai Chi may help strengthen self−care awareness, emotional regulation, and perceived social support.

The improvement in exercise behavior was especially pronounced and can be attributed directly to structured, supervised Tai Chi practice. Regular group sessions likely enhanced participants’ exercise skills, confidence, and endurance, and may have facilitated the formation of more stable exercise habits, addressing common barriers such as low motivation and weak self−management in adolescent PCOS patients (59). The mind-body characteristics of Tai Chi, including focused attention on breathing and movement, may alleviate anxiety and improve mood, thereby mitigating psychological distress related to PCOS symptoms such as acne, hirsutism, and menstrual irregularities. The group−based format may foster peer support and reduce feelings of isolation. This is particularly relevant for young women who may experience PCOS−related stigma or body image concerns (40).

### 4.4 Exploratory subgroup analyses: hypothesis−generating observations

A distinctive feature of this study was the inclusion of pre−specified subgroup analyses to explore potential effect modifiers. For health−promoting lifestyle score, intervention effects were broadly consistent across BMI strata (<25 vs ≥25 kg/m²; interaction P = 0.089) and age groups (interaction P = 0.687), although point estimates suggested slightly larger gains in participants with higher baseline BMI. In contrast, BMI reduction showed clear evidence of effect modification by baseline BMI, with a significant interaction (P<0.001): participants with lower baseline BMI experienced greater additional weight loss from the combined intervention than from diet alone, whereas in those with higher baseline BMI, both groups achieved similar reductions. These findings indicate that, at least in this sample, the incremental benefit of Tai Chi for weight reduction was more obvious among participants who were not yet obese, although dietary intervention alone still produced meaningful weight loss in overweight and obese participants. This pattern should be interpreted cautiously and does not imply that Tai Chi is unnecessary for higher BMI patients, given its benefits on other outcomes.

​Baseline menstrual cycle severity emerged as a more robust effect modifier. Participants with severe menstrual irregularities (>60 days) derived greater benefit from the combined intervention than those with moderate irregularities (≤60 days) for both lifestyle and menstrual outcomes. The between−group difference in lifestyle score improvement was larger in the severe subgroup (19.39 vs 12.30 points; interaction P = 0.042), and the between−group difference in menstrual cycle reduction was also greater (12.61 vs 5.07 days; interaction P = 0.028). These patterns suggest that patients with more pronounced menstrual dysfunction may experience relatively larger gains from the combined intervention. A trend toward effect modification was also observed for testosterone reduction (interaction P = 0.068), with larger between−group differences in the severe subgroup, although this result should be regarded as exploratory.

Overall, these subgroup findings have potential implications for more individualized care. The subgroup data tentatively suggest that patients with severe menstrual irregularities might derive relatively greater benefit, but this observation is hypothesis−generating and requires confirmation in adequately powered studies before it can inform clinical decision−making. At the same time, the subgroup analyses were based on modest sample sizes, and some interaction tests did not reach conventional significance thresholds, so these observations require confirmation in larger, adequately powered studies.

### 4.5 Robustness of findings

Multiple sensitivity analyses were conducted to evaluate the robustness of the primary findings. Excluding statistical outliers identified by Z−scores for the primary outcome did not materially change the between−group differences, and all results remained highly significant (P<0.001). Covariate−adjusted analyses using ANCOVA, controlling for baseline lifestyle score, age, and BMI, yielded slightly larger effect estimates than the unadjusted analyses, again with P<0.001. Per−protocol analyses restricted to participants with higher BMI or meeting adherence criteria produced estimates similar in magnitude and direction to the complete−case analyses. The ITT−LOCF analysis (**Table 1**) further confirmed these findings: the primary outcome remained highly significant (MD = 10.90, P < 0.001, d = 0.64), and all outcomes maintained the same direction of effect, although effect sizes were attenuated as expected under the conservative LOCF assumption.

Across all sensitivity approaches, the combined intervention consistently showed greater improvements in health−promoting lifestyle score, BMI, menstrual cycle duration, and testosterone levels than the control condition, whereas dietary nutrition scores were consistently similar between groups. This convergence of findings across analytic strategies supports the internal consistency and robustness of the conclusions.

### 4.6 Comparison with previous literature

The present findings are broadly consistent with and extend prior research on mind-body exercise in metabolic and reproductive disorders (37, 60). A limited number of studies have examined Tai Chi or similar practices in women with PCOS, and most have been small or non−randomized (36, 39). This trial contributes evidence from one of the first randomized controlled studies examining combined Tai Chi and dietary intervention specifically in adolescent PCOS patients.

Lifestyle intervention trials in PCOS typically report small−to−medium effects on metabolic and reproductive outcomes (13, 51). In contrast, the current study observed very large effect sizes for health−promoting lifestyle outcomes and medium or small−to−medium effects for physiological indicators. The comparatively large behavioral effects may reflect the integrated nature of the intervention, which simultaneously targets physical activity, stress reduction, and social support. Nevertheless, the relatively small sample size and single−center design suggest that caution is warranted when generalizing the magnitude of effect, and replication in larger and more diverse populations is needed.

When considered alongside pharmacological options, the clinical impact of the combined intervention appears encouraging. For example, prior studies report numbers needed to treat in the range of 4–8 for metformin in improving menstrual regularity in PCOS (13, 61, 62). In our study, the NNT for cycle normalization was 3.9, and the NNT for achieving substantial lifestyle score improvement was even lower. While direct comparisons between behavioral and pharmacological interventions should be made cautiously, the findings suggest that Tai Chi combined with dietary adjustment may offer benefits comparable to first−line medications for some outcomes. This combined approach carries minimal side effects and produces broader improvements in lifestyle and psychosocial health.

### 4.7 Clinical implications

This trial has several implications for clinical practice and public health. First, these preliminary findings suggest that Tai Chi may be a feasible and potentially beneficial adjunctive exercise option for adolescents and young adults with PCOS who prefer non−pharmacological approaches, although confirmation in multi−center trials is needed before integration into clinical management programs can be recommended. Second, Tai Chi does not require specialized equipment and can be practiced in a range of settings, including university campuses, community centers, parks, and home environments, which enhances feasibility and scalability. Group−based Tai Chi instruction is relatively low cost, suggesting potential for favorable cost−effectiveness, although formal economic evaluations are needed. Third, the intervention addresses multiple domains-physical fitness, health−promoting lifestyle behaviors, psychological well−being, and social functioning-which may better match the complex needs of PCOS patients than interventions focused on a single target. Fourth, exploratory subgroup data tentatively suggest possible differential benefit by menstrual severity, but these observations require replication before they can guide clinical prioritization. Finally, the gentle, non−competitive nature of Tai Chi may be especially acceptable to adolescent girls with limited exercise experience or negative perceptions of traditional sports, and its meditative aspects may appeal to young adults seeking stress management alongside physical health benefits.

### 4.8 Strengths and limitations

This study has several strengths. It employed a randomized controlled design with successful randomization verified by baseline comparability, and used a 6−month intervention period considered adequate for assessing sustained lifestyle changes. The outcomes covered a broad range of domains, including lifestyle behaviors, anthropometric indices, menstrual characteristics, and hormonal measures, providing a comprehensive picture of intervention effects. The primary outcome was assessed with a validated health−promoting lifestyle scale specific to PCOS, and standardized procedures were used for anthropometric and laboratory measurements. Pre−specified subgroup analyses provided insight into potential effect modifiers, and multiple sensitivity analyses supported the robustness of the main findings. Finally, effect sizes, NNTs, and MCID comparisons were reported to facilitate clinical interpretation.

Several limitations should also be acknowledged. The sample size was modest (86 completers) and drawn from a single university in Zhengzhou, which may limit statistical power for some subgroup analyses and restrict generalizability. Attrition was substantial (28.3% in each group), and although dropout rates were balanced, participants who completed the study may have been more motivated or health−conscious than non−completers. Blinding of participants and instructors was not feasible, which may have introduced performance and reporting bias, although outcome assessors and laboratory staff were blinded to group allocation.

Importantly, the absence of an active exercise control group (e.g., aerobic exercise or group walking of equivalent frequency and duration) precludes attribution of the observed benefits specifically to Tai Chi. The improvements may reflect general effects of increased physical activity, social engagement during group sessions, or the structured support environment, rather than Tai Chi−specific therapeutic mechanisms.

The trial was not prospectively registered in a clinical trial registry, which does not meet current Recommendations for the Conduct, Reporting, Editing, and Publication of Scholarly Work in Medical Journals (ICMJE recommendations) for clinical trial reporting and constitutes a methodological limitation.

The exceptionally large effect sizes for exercise and total lifestyle score (Cohen’s d > 2.5) should be interpreted with caution. Because participants in the intervention group attended supervised Tai Chi sessions five times per week, improvements in exercise scores partly reflect adherence to the intervention protocol itself rather than independently developed exercise habits. This inherent overlap between intervention delivery and outcome measurement is a common feature of supervised exercise trials but limits the ability to distinguish structured participation from durable behavioral change.

Relatedly, the exercise dimension of the Health−Promoting Lifestyle Scale assesses general exercise behaviors without specifically naming Tai Chi, but participants in the intervention group likely incorporated their Tai Chi practice when responding to items about regular exercise and aerobic activity. This measurement overlap means that the exercise subscale effect size partly reflects the intervention itself rather than independently acquired exercise habits, and the total lifestyle score is affected to the extent that it includes this subscale. Future studies should consider outcome measures independent of the specific intervention, or include supplementary objective measures of physical activity (e.g., accelerometry).

The study sample was limited to female college students aged 18–22 years at a single university in China, a treatment-naïve population with cultural familiarity with Tai Chi and access to campus infrastructure (gymnasium, outdoor plazas, structured academic schedule) that facilitated high−frequency session attendance. The intervention intensity (60 minutes, 5 sessions per week) may not be replicable in routine clinical practice or among non−student populations with competing work and family obligations. The generalizability of findings to older women, different ethnicities, non−Chinese cultural contexts, and clinical settings with fewer resources for supervised group exercise remains to be established.

Aggregate Tai Chi session attendance rates were not systematically quantified, limiting the ability to report dose−response relationships. Dietary adherence was assessed through counseling−based self−report rather than objective measures such as validated food frequency questionnaires or dietary biomarkers, reducing the ability to verify compliance with the dietary protocol.

In addition, only total testosterone was measured as a hormonal endpoint; other markers critical to understanding PCOS pathophysiology — including fasting insulin, HOMA−IR, LH, FSH, LH/FSH ratio, SHBG, free androgen index, and inflammatory markers — were not assessed. This substantially limits the ability to draw mechanistic conclusions about the pathways through which Tai Chi may influence endocrine and metabolic function in PCOS. The 6−month follow−up period does not address long−term maintenance of lifestyle changes or sustainability of Tai Chi practice. Exercise intensity was monitored using subjective RPE rather than objective measures such as heart rate monitoring, and dietary adherence was assessed primarily through counseling and self−report. Finally, the study focused on intermediate outcomes such as lifestyle behaviors, BMI, menstrual cycle, and testosterone, rather than definitive endpoints such as ovulation rates, pregnancy, or long−term metabolic disease, so the implications for these ultimate outcomes remain to be established.

### 4.9 Future research directions

Future research should build on these findings in several ways. Multi−center trials with larger, more diverse samples are needed to confirm the effects of Tai Chi−based interventions across different regions, ethnicities, and clinical phenotypes of PCOS. Longer follow−up periods (12–24 months or more) would help clarify the durability of behavioral and clinical changes, and the sustainability of Tai Chi practice after the end of supervised intervention.

Critically, future trials should incorporate active exercise control groups matched for frequency, duration, and social contact to determine whether Tai Chi offers specific advantages beyond general physical activity for PCOS management.

Future trials should also incorporate objective monitoring of exercise attendance (e.g., electronic sign−in systems with automated reporting) and dietary adherence (e.g., validated food frequency questionnaires, dietary biomarkers, or systematic food diary analysis) to enable dose−response analyses and verify compliance with intervention protocols.

Comprehensive hormonal and metabolic profiling, including LH, FSH, insulin, HOMA−IR, SHBG, and free androgen index, would allow more detailed exploration of mechanisms underlying menstrual and androgenic improvements. Comparisons of different Tai Chi styles, practice frequencies, and session durations could identify optimal training parameters for PCOS management. Cost−effectiveness analyses would inform resource allocation and policy decisions regarding the integration of Tai Chi into clinical guidelines.

Objective monitoring of exercise intensity (e.g., heart rate, accelerometry) and dietary adherence (food records, biomarkers) should be incorporated to better characterize dose-response relationships. Qualitative research exploring participants’ experiences, barriers, facilitators, and preferences could help optimize intervention design and implementation. Trials combining Tai Chi with pharmacological therapies may clarify whether Tai Chi can enhance treatment response or facilitate medication dose reduction. Finally, mechanistic studies focusing on inflammatory markers, oxidative stress, autonomic function (e.g., heart rate variability), and hypothalamic-pituitary-ovarian axis regulation, as well as long−term reproductive outcomes such as ovulation and pregnancy rates, are warranted.

## 5 Conclusion

In conclusion, this single−center randomized controlled trial provides preliminary evidence that Tai Chi exercise combined with dietary adjustment may be more effective than dietary intervention alone in improving health−promoting lifestyle behaviors in female college students with PCOS. The primary outcome showed a large between−group difference in health−promoting lifestyle score (d = 2.62), although the behavioral effect sizes partly reflect supervised session participation. Secondary outcomes showed modest improvements in BMI, menstrual regularity, and testosterone levels favoring the combined intervention, but the study was not independently powered for these endpoints.

Key findings include: (1) large standardized effect sizes for the health−promoting lifestyle score and exercise behavior; (2) significant between−group differences in BMI reduction and menstrual cycle shortening, with a significant within−group reduction in testosterone only in the Tai Chi group; (3) exploratory subgroup data suggesting, but not confirming, that patients with more severe menstrual irregularities might derive comparatively greater benefit; and (4) consistent advantages of the combined intervention across multiple sensitivity analyses and several clinically meaningful response thresholds.

As a safe, low−cost, and accessible modality, Tai Chi combined with dietary adjustment may serve as a useful, safe, and accessible adjunctive exercise modality within lifestyle−based management for young women with PCOS. However, in the absence of an active exercise comparator, the specific contribution of Tai Chi versus general physical activity cannot be determined. Multi−center trials with active exercise comparators, comprehensive metabolic profiling, objective adherence monitoring, and long−term follow−up are needed before definitive clinical recommendations can be made.

## Data Availability

The raw data supporting the conclusions of this article will be made available by the authors, without undue reservation.
